# High diversity of dietary flavonoid intake is associated with a lower risk of all-cause mortality and major chronic diseases

**DOI:** 10.1038/s43016-025-01176-1

**Published:** 2025-06-02

**Authors:** Benjamin H. Parmenter, Alysha S. Thompson, Nicola P. Bondonno, Amy Jennings, Kevin Murray, Aurora Perez-Cornago, Jonathan M. Hodgson, Anna Tresserra-Rimbau, Tilman Kühn, Aedín Cassidy

**Affiliations:** 1 Nutrition & Health Innovation Research Institute, School of Medical and Health Sciences, Edith Cowan University, Joondalup, Western Australia Australia; 2 Co-Centre for Sustainable Food Systems & Institute for Global Food Security, Queen’s University Belfast, Belfast, UK; 3 Danish Cancer Society Research Centre, Copenhagen, Denmark; 4 School of Population and Global Health, University of Western Australia, Crawley, Western Australia Australia; 5 Cancer Epidemiology Unit, Nuffield Department of Population Health, University of Oxford, Oxford, UK; 6 Department of Nutrition, Food Science and Gastronomy, XIA, School of Pharmacy and Food Sciences, INSA, University of Barcelona, Barcelona, Spain; 7 Centro de Investigación Biomédica en Red Fisiopatología de la Obesidad y la Nutrición, Madrid, Spain; 8 Department of Nutritional Sciences, University of Vienna, Vienna, Austria; 9 Center for Public Health, Medical University of Vienna, Vienna, Austria

**Keywords:** Epidemiology, Nutrition, Diseases

## Abstract

Higher habitual intakes of dietary flavonoids have been linked with a lower risk of all-cause mortality and major chronic disease. Yet, the contribution of diversity of flavonoid intake to health outcomes remains to be investigated. Here, using a cohort of 124,805 UK Biobank participants, we show that participants who consumed the widest diversity of dietary flavonoids, flavonoid-rich foods and/or specific flavonoid subclasses had a 6–20% significantly lower risk of all-cause mortality and incidence of cardiovascular disease, type 2 diabetes, cancer, respiratory disease and neurodegenerative disease. Furthermore, we report that both quantity and diversity of flavonoids are independent predictors of mortality and several chronic diseases, suggesting that consuming a higher quantity and wider diversity is better for longer-term health than either component alone. These findings suggest that consuming several different daily servings of flavonoid-rich foods or beverages, such as tea, berries, apples, oranges or grapes, may lower risk of all-cause mortality and chronic disease.

## Abstract (web summary)

The intake of polyphenolic compounds is part of a healthy diet, but the contribution of a diversity of flavonoids to health outcomes remains unknown. This study shows that participants in the UK Biobank cohort with the greatest diversity of flavonoid intake had a 6–20% lower risk of all-cause mortality and major chronic diseases.

## Main

Flavonoids are (poly)phenolic compounds that occur abundantly in the human diet^1^. Sources are quite diverse, ranging from fruits and vegetables to nuts and legumes, as well as wines and teas^2^. A wide range of flavonoids are found in foods and beverages, and these can be classified into several subclasses including flavonols, anthocyanins, flavan-3-ols, flavanones and flavones^1^. Following their consumption and absorption, flavonoids—through their downstream metabolites—have the potential to improve human health^1^. Since the early 1990s^3^, numerous prospective cohort studies have observed that a higher habitual consumption of several flavonoid subclasses is associated with a lower risk of all-cause mortality^4–6^, cardiovascular disease (CVD)^2,7^, type 2 diabetes (T2DM)^8,9^, cancer^10^, respiratory disease^11^ and neurodegenerative disease^12,13^. Due to variations in their chemical structure, bioavailability and metabolism, different flavonoid compounds exert a range of biological effects^14^. Among these, some of their most widely recognized activities include anti-inflammatory and antioxidative stress effects, which are fundamental mechanisms underlying the development and progression of many chronic diseases^15^. Additionally, flavonoids exhibit more specific protective functions, including promoting endothelial integrity and function^16^, crucial for cardiovascular health, and anti-senescence effects^17^ that may delay age-related tissue deterioration, in addition to antiproliferative activities^18^ that contribute to cancer prevention. These represent just some examples of the many mechanisms through which flavonoids exert their beneficial effects across diverse chronic conditions^1,15^.

Because different flavonoid compounds can exert different biological benefits, we hypothesized that consuming a higher diversity of dietary flavonoids may afford better health protection than consuming a low diversity of flavonoids. However, to date, no prospective studies have considered the impact of consuming a higher diversity of dietary flavonoids on the risk of all-cause mortality or major chronic disease. In several research fields, including in the assessment of gut microbial diversity^19–21^, the diversity of a system can be calculated using Shannon’s equation for entropy^22^ converted to Hill’s effective numbers^23,24^. Using this approach, we can determine the diversity of flavonoid intake, accounting for both the variation (or number of different flavonoids consumed) and their distribution of intake (wherein those flavonoids consumed in smaller amounts relative to others are weighted less). The aims of this study, therefore, were: (1) to estimate diversity of flavonoid intake across levels of total dietary flavonoids, individual flavonoid subclasses and flavonoid-rich foods, and then examine their associations with the risk of all-cause mortality and incidence of chronic disease including CVD, T2DM, total cancer, respiratory disease and neurodegenerative disease; and (2) to assess the potential benefits of consuming both a higher quantity and a wider diversity of flavonoid intake on the risk of these outcomes in participants from the UK Biobank.

## Results

### Cohort characteristics

**Fig. 1:**
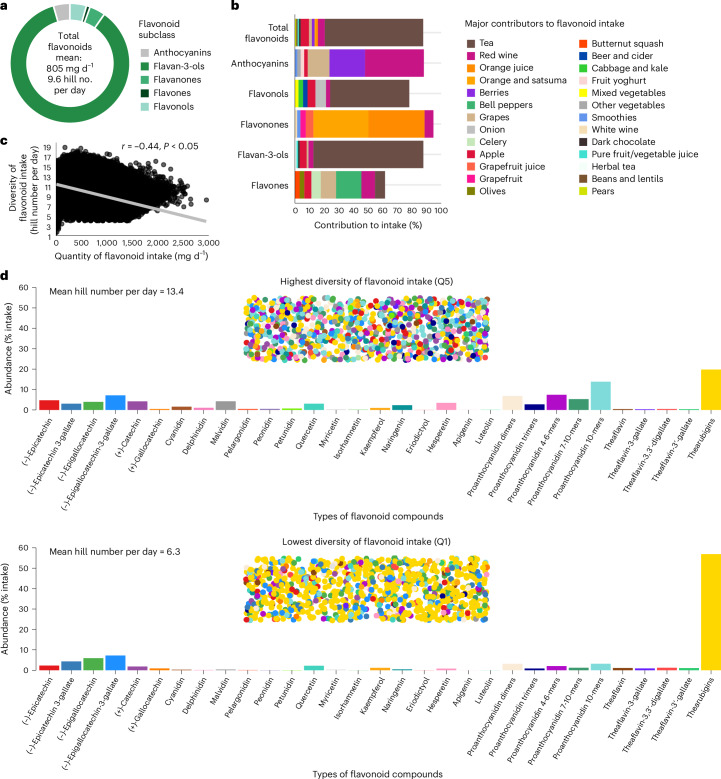
Flavonoid intake in the UK Biobank. **a**, Composition of flavonoid intake. **b**, Major dietary contributors to flavonoid intake, showing the topmost contributors to intake only; blank spaces up to 100% represent other smaller contributors that are not shown. **c**, Two-sided Pearson correlation between quantity and diversity of flavonoid intake. **d**, Diversity of flavonoid consumption among participants with the most (Q5) and least (Q1) diverse intakes. In **d**, the bar charts are matched for quantity of flavonoid intake (1,000 mg d^−1^) and show the average abundance (% intake) of each flavonoid per day. The dotted areas represent each diet, where each circle is an individual flavonoid and each colour is a different flavonoid (corresponding to the colours and distribution on the bar charts). Data from participants with ≥2 Oxford WebQ dietary questionnaires (*n* = 124,805).

In this cohort of 124,805 UK adults, aged ≥40 yr (median [Q1–Q3], 60.2 [53.0–65.2] yr; Q, quintile), ~56% (*n* = 69,674) were female and most were non-smokers (>90%; *n* = 115,961) (Table 1). Around 60% (*n* = 75,111) of participants were either overweight or obese (Table 1). At baseline, ~4% (*n* = 5,162) had diabetes (type 1 or 2), ~25% (*n* = 32,877) were hypertensive and ~15% (*n* = 19,827) had high cholesterol. Over a range of 8.7–10.6 median years of follow-up for the different outcomes (maximum, 11.8 yr), there were 5,780 deaths, 6,920 CVD cases, 3,421 T2DM cases, 9,441 cancer cases, 12,945 respiratory disease cases and 1,921 cases of neurodegenerative disease. Participants had a median flavonoid intake of 792 mg d^−1^ (range, 0.05–3,611 mg d^−1^), which was comprised of a wide diversity of an effective (Hill) number of 9.4 flavonoid types per day (range, 1.8–19.0) (Fig. 1). Flavan-3-ols were the main subclass contributing to total flavonoid intake, accounting for 87% of consumption. Anthocyanins, flavonols and flavanones each contributed ~4.5% of total flavonoid intake; <1% was from flavones. Tea (black and green) was the main source of total flavonoid intake (67%), followed by apples (5.8%), red wine (4.7%), grapes (1.9%), berries (1.9%), dark chocolate (1.2%), oranges and satsumas (1.1%) and orange juice (1.1%), which collectively comprised ~85% of total intake; numerous other food sources contributed to the remaining intake (Fig. 1 and Supplementary Table 1). Overall, those with a higher quantity of flavonoid intake tended to have a lower diversity (*r* = −0.44), although this varied for individual subclasses (Fig. 1 and Supplementary Table 2). Compared to participants with the lowest diversity, those with the highest diversity had a better distribution of flavonoid intake, consuming more anthocyanins (for example, malvidin, cyanidin), flavanones (for example, hesperidin, naringenin) and proanthocyanidins (for example, dimers to polymers) relative to thearubigin, a compound derived exclusively from tea, and which dominated intake in those with the least diverse consumption (Fig. 1 and Supplementary Table 3). Analysis of flavonoid-rich foods showed those with the lowest diversity consumed mostly tea, and those with the highest diversity consumed relatively more berries, apples, grapes, red wine and oranges (Supplementary Table 4). Those with the highest flavonoid diversity were more likely to be female, older, have a lower body mass index (BMI), be more physically active and have a higher education and were less likely to be current smokers (Table 1).

**Table 1:** Baseline characteristics of study population

	Diversity of flavonoid intake					
	Total population (*n* = 124,805)	Q1 (*n* = 24,961)	Q2 (*n* = 24,961)	Q3 (*n* = 24,961)	Q4 (*n* = 24,961)	Q5 (*n* = 24,961)
Demographic and lifestyle characteristics						
Sex (female)	69,674 (55.8%)	13,666 (54.7%)	14,103 (56.5%)	13,855 (55.5%)	13,752 (55.1%)	14,298 (57.3%)
Age (years)	60.2 [53.0–65.2]	59.2 [52.1–64.7]	60.2 [53.1–65.2]	60.2 [53.0–65.3]	60.6 [53.3–65.4]	60.7 [53.3–65.4]
BMI (kg m^−^^2^)						
Underweight (<18.5)	719 (0.6%)	131 (0.5%)	152 (0.6%)	153 (0.6%)	148 (0.6%)	135 (0.5%)
Healthy weight (18.5 to <25)	48,693 (39.0%)	8,922 (35.7%)	9,765 (39.1%)	9,883 (39.6%)	9,988 (40.0%)	10,135 (40.6%)
Overweight (25 to <30)	50,833 (40.7%)	10,281 (41.2%)	10,216 (40.9%)	10,077 (40.4%)	10,105 (40.5%)	10,154 (40.7%)
Obese (≥30)	24,278 (19.5%)	5,559 (22.3%)	4,767 (19.1%)	4,796 (19.2%)	4,669 (18.7%)	4,487 (18.0%)
Ethnicity						
Asian	1,463 (1.2%)	235 (0.9%)	295 (1.2%)	346 (1.4%)	331 (1.3%)	256 (1.0%)
Black	966 (0.8%)	112 (0.4%)	152 (0.6%)	203 (0.8%)	231 (0.9%)	268 (1.1%)
Mixed	668 (0.5%)	105 (0.4%)	107 (0.4%)	141 (0.6%)	147 (0.6%)	168 (0.7%)
Other	723 (0.6%)	93 (0.4%)	108 (0.4%)	158 (0.6%)	175 (0.7%)	189 (0.8%)
White	120,569 (96.6%)	24,336 (97.5%)	24,217 (97.0%)	24,020 (96.2%)	23,994 (96.1%)	24,002 (96.2%)
Smoking status						
Current	8,577 (6.9%)	2,375 (9.5%)	1,521 (6.1%)	1,471 (5.9%)	1,574 (6.3%)	1,636 (6.6%)
Never	71,321 (57.1%)	14,185 (56.8%)	14,901 (59.7%)	14,655 (58.7%)	14,149 (56.7%)	13,431 (53.8%)
Previous	44,640 (35.8%)	8,346 (33.4%)	8,489 (34.0%)	8,781 (35.2%)	9,180 (36.8%)	9,844 (39.4%)
Alcohol intake (g d^−1^)	10.0 [2.6–20.0]	7.1 [0.3–17.143]	8.6 [1.4–17.1]	8.6 [2.3–18.6]	11.4 [2.9–20.0]	12.9 [5.7–24.3]
MET-h per week	19.6 [8.5–38.9]	16.8 [6.8–36.4]	19.1 [8.2–38.4]	19.9 [9.0–39.0]	20.55 [9.2–39.6]	21.4 [10.0–40.6]
Education						
Low	17,122 (13.7%)	4,410 (17.7%)	3,583 (14.4%)	3,275 (13.1%)	3,065 (12.3%)	2,789 (11.2%)
Medium	21,180 (17.0%)	5,078 (20.3%)	4,418 (17.7%)	4,078 (16.3%)	3,969 (15.9%)	3,637 (14.6%)
High	78,015 (62.5%)	12,850 (51.5%)	15,121 (60.6%)	16,053 (64.3%)	16,568 (66.4%)	17,423 (69.8%)
Townsend deprivation index	−1.6 (2.8)	−1.6 (2.9)	−1.8 (2.7)	−1.7 (2.8)	−1.6 (2.8)	−1.5 (2.9)
Medical history						
Hypertensive	32,877 (26.3%)	6,972 (27.9%)	6,531 (26.2%)	6,508 (26.1%)	6,423 (25.7%)	6,443 (25.8%)
Hypercholesterolaemic	19,827 (15.9%)	4,059 (16.3%)	3,913 (15.7%)	3,937 (15.8%)	3,918 (15.7%)	4,000 (16.0%)
Diabetes (type 1 or 2)	5,162 (4.1%)	1,164 (4.7%)	1,061 (4.3%)	1,051 (4.2%)	983 (3.9%)	903 (3.6%)
Dietary characteristics						
Energy (kJ d^−1^)	8,397.0 [7,177.3–9,753.4]	8,051.4 [6,842.5–9,364.9]	8,361.1 [7,170.5–9,699.1]	8,506.6 [7,301.8–9,857.6]	8,543.4 [7,308.1–9,909.5]	8,532.8 [7,295.0–9,923.2]
Total flavonoids (mg d^−1^)	792.3 [451.4–1,118.6]	1,100.8 [833.7–1,347.3]	976.5 [691.8–1,251.1]	800.8 [455.8–1,087.7]	608.8 [330.6–906.6]	491.6 [322.8–714.4]
Total flavonoid (Hill number per day)	9.4 [7.6–11.5]	6.4 [5.8–6.9]	8.0 [7.6–8.4]	9.4 [9.1–9.8]	11.0 [10.6–11.5]	13.1 [12.5–14.1]
Flavonoid-rich food (Hill number per day)	2.7 [1.9–3.6]	1.6 [1.3–2.0]	2.4 [1.9–2.8]	2.9 [2.3–3.5]	3.4 [2.6–4.1]	3.8 [2.9–4.8]
Flavan-3-ols (mg d^−1^)	706.1 [374.2–1,020.3]	1,032.1 [781.9–1,268.8]	898.8 [632.0–1,152.3]	716.6 [397.1–980.4]	528.5 [257.1–797.4]	392.3 [240.8–589.6]
Anthocyanins (mg d^−1^)	20.5 [7.1–40.2]	5.5 [2.3–14.4]	16.0 [6.2–30.1]	21.5 [8.6–39.0]	28.5 [13.5–49.5]	40.5 [23.3–63.3]
Flavanols (mg d^−1^)	31.7 [20.2–43.5]	41.3 [31.8–49.9]	37.5 [27.2–48.0]	31.6 [19.8–42.7]	25.8 [15.9–36.9]	22.9 [16.0–31.8]
Flavanones (mg d^−1^)	17.9 [5.3–35.7]	6.7 [1.3–20.4]	14.8 [4.1–31.2]	18.9 [6.4–36.3]	21.6 [8.5–39.6]	26.9 [12.9–44.4]
Flavones (mg d^−1^)	0.9 [0.5–1.4]	0.5 [0.2–0.8]	0.8 [0.4–1.2]	0.9 [0.5–1.5]	1.1 [0.6–1.7]	1.2 [0.8–1.8]
Red/processed meat (servings per day)	0.8 [0.3–1.3]	0.9 [0.4–1.5]	0.8 [0.3–1.3]	0.7 [0.3–1.3]	0.7 [0.3–1.3]	0.7 [0.3–1.3]
Whole grains (servings per day)	2.0 (1.0–3.0)	1.67 (1.0–2.8)	2.0 (1.0–3.0)	2.0 (1.0–3.0)	2.0 (1.0–3.0)	2.0 (1.0–3.0)
Refined grains (servings per day)	0.8 [0.3–1.5]	1.0 [0.5–2.0]	0.8 [0.3–1.5]	0.8 [0.3–1.5]	0.7 [0.3–1.4]	0.7 [0.3–1.3]
Coffee intake (cups per day)	0.0 [0.0–1.0]	0.0 [0.0–1.0]	0.0 [0.0–1.0]	0.0 [0.0–1.0]	0.0 [0.0–1.0]	0.0 [0.0–1.0]
Sugary drinks (servings per day)	0.1 [0.0–0.7]	0.2 [0.0–0.7]	0.2 [0.0–0.7]	0.2 [0.0–0.7]	0.2 [0.0–0.7]	0.0 [0.0–0.7]
Saturated fat (g d^−1^)	25.7 [19.7–32.7]	25.9 [19.9–32.9]	26.1 [20.1–33.2]	26.0 [19.9–33.2]	25.4 [19.5–32.5]	24.8 [18.9–31.7]
Sodium (g d^−1^)	1.9 [1.5–2.3]	1.9 [1.5–2.3]	1.9 [1.5–2.3]	1.9 [1.5–2.3]	1.8 [1.5–2.3]	1.8 [1.5–2.3]

Data are expressed as mean (±s.d.), median [Q1–Q3] or *n* (%), unless otherwise stated. Relative frequencies (%) may not equate to 100% due to missing values.

### Total flavonoids, all-cause mortality and chronic disease

**Fig. 2:**
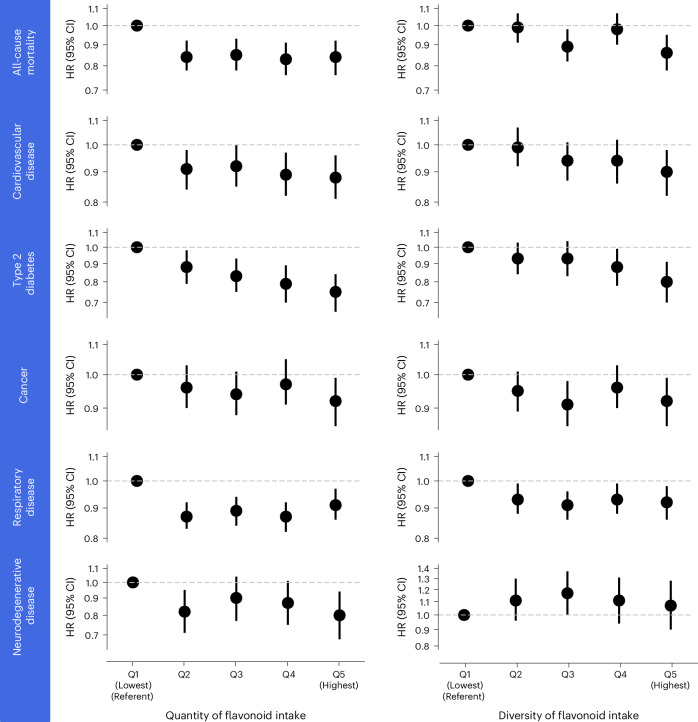
Quantity and diversity of dietary flavonoid intake and risk of all-cause mortality and chronic disease. HRs (95% CI) for all-cause mortality and major chronic disease according to the quantity and diversity of dietary flavonoid intake (in quintiles). HRs are from Cox proportional-hazards models using age as the underlying timescale. Quantity of flavonoid intake is mutually adjusted for diversity of flavonoid intake and vice versa. Further adjustments are made for covariates in model 5 including sex, region of residence, number of dietary assessments, BMI, smoking status, physical activity, alcohol intake, education, ethnicity, socioeconomic status plus intakes of red and processed meat, refined grains, whole grains, sugary drinks, coffee, saturated fatty acids, sodium and dietary energy, and history of diabetes (type 1 or 2; not adjusted in T2DM analysis), hypertension and hypercholesterolaemia and for analysis of all-cause mortality, further adjustments were made for prevalent CVD, cancer, respiratory disease, and neurodegenerative disease at baseline. Corresponding sample sizes, event rates and additional details are provided in Table 2.

Following mutual adjustment, and after accounting for sociodemographic, lifestyle, dietary and medical risk factors, both the quantity and diversity of total dietary flavonoid intake were independently associated with a lower risk of all-cause mortality and several chronic diseases (model 5; Fig. 2). Holding the quantity of flavonoid intake constant, participants with the highest (compared to lowest) diversity (Q5 versus Q1), characterized as consuming an additional 6.7 effective flavonoid types per day, had a 14% lower risk of all-cause mortality (hazard ratio (HR) (95% confidence interval (CI)), 0.86 (0.78, 0.95)), a 10% lower risk of CVD (0.90 (0.82, 0.98)), a 20% lower risk of T2DM (0.80 (0.70, 0.91)), an 8% lower risk of total cancer (0.92 (0.85, 0.99)) and an 8% lower risk of respiratory disease (0.92 (0.86, 0.98)); no association was observed for neurodegenerative disease (model 5; Table 2 and Fig. 2). For quantity of flavonoid intake, when holding diversity constant, participants in the second quintile (median intake, ~500 mg d^−1^), were at a 16% (0.84 (0.78, 0.92)), 9% (0.91 (0.84, 0.98)), 12% (0.88 (0.79, 0.98)) and 13% (0.87 (0.83, 0.92)) lower risk of all-cause mortality, CVD, T2DM and respiratory disease, respectively, compared with those in Q1 (median intake, ~230 mg d^−1^ (model 5; Table 2)). At higher levels of exposure, these HRs remained relatively constant, except for T2DM, for which the lowest risks were observed for those in Q5 (0.75 (0.66, 0.84)). The lowest risks for cancer and neurodegenerative diseases were seen in Q5 (median intake, ~1,400 mg d^−1^), reaching an 8% (0.92 (0.85, 0.99)) and 20% (0.80 (0.68, 0.94)) lower disease risk, respectively, compared with Q1 (model 5; Table 2 and Fig. 2). In general, progressive adjustment for participant demographics (model 2), lifestyle (model 3), dietary (model 4) and medical risk factors (model 5) attenuated, but did not materially alter, the associations (Table 2). We then tested for interactions between quantity and diversity of flavonoid intake (across the aforementioned outcomes), and although no interactions were observed (*P*_interaction_ all >0.05 (model 5)), the independent prediction of both quantity and diversity of flavonoid intake with all-cause mortality and several chronic diseases still suggests that higher intakes of both is associated with greater disease risk reduction compared with higher intakes of either aspect alone.

**Table 2:** Quantity and diversity of dietary flavonoid intake associate with risk of all-cause mortality and incidence of major chronic disease

	Quantity of total dietary flavonoid intake					Diversity of total dietary flavonoid intake				
	Q1	Q2	Q3	Q4	Q5	Q1	Q2	Q3	Q4	Q5
**All-cause mortality**										
Population	24,961	24,961	24,961	24,961	24,961	24,961	24,961	24,961	24,961	24,961
Number of events	1,268 (5.1%)	1,072 (4.3%)	1,117 (4.5%)	1,131 (4.5%)	1,192 (4.8%)	1,249 (5.0%)	1,190 (4.8%)	1,079 (4.3%)	1,211 (4.9%)	1,051 (4.2%)
Intake	234 [0.1–378]	526 [378–665]	792 [665–915]	1,048 [915–1,197]	1,400 [1,197–3,611]	6.4 [1.8–7.3]	8.0 [7.3–8.7]	9.4 [8.7–10.2]	11.0 [10.2–11.9]	13.1 [11.9–19.0]
HR (95% CI)										
Model 1 (minimally adjusted)	Ref.	**0.76 (0.70, 0.83)**	**0.72 (0.67, 0.79)**	**0.69 (0.63, 0.75)**	**0.70 (0.64, 0.76)**	Ref.	**0.89 (0.82, 0.97)**	**0.77 (0.71, 0.84)**	**0.82 (0.76, 0.90)**	**0.70 (0.64, 0.77)**
Model 2 (+ demographics)	Ref.	**0.80 (0.73, 0.87)**	**0.77 (0.71, 0.84)**	**0.74 (0.68, 0.81)**	**0.76 (0.69, 0.83)**	Ref.	0.93 (0.86, 1.01)	**0.82 (0.75, 0.89)**	**0.88 (0.81, 0.96)**	**0.76 (0.69, 0.83)**
Model 3 (+ lifestyle)	Ref.	**0.83 (0.76, 0.90)**	**0.82 (0.76, 0.89)**	**0.80 (0.74, 0.88)**	**0.81 (0.74, 0.88)**	Ref.	0.98 (0.90, 1.06)	**0.88 (0.81, 0.96)**	0.96 (0.88, 1.05)	**0.84 (0.76, 0.92)**
Model 4 (+ diet)	Ref.	**0.84 (0.78, 0.92)**	**0.85 (0.78, 0.92)**	**0.83 (0.76, 0.91)**	**0.84 (0.77, 0.92)**	Ref.	0.99 (0.91, 1.07)	**0.89 (0.82, 0.97)**	0.98 (0.90, 1.07)	**0.87 (0.79, 0.96)**
Model 5 (+ medical history)	Ref.	**0.84 (0.78, 0.92)**	**0.85 (0.78, 0.93)**	**0.83 (0.76, 0.91)**	**0.84 (0.76, 0.92)**	Ref.	0.99 (0.91, 1.07)	**0.89 (0.82, 0.98)**	0.98 (0.90, 1.07)	**0.86 (0.78, 0.95)**
**CVD incidence**										
Population	23,801	23,801	23,800	23,801	23,801	23,801	23,801	23,800	23,801	23,801
Number of events	1,412 (5.9%)	1,305 (5.5%)	1,385 (5.8%)	1,392 (5.8%)	1,426 (6.0%)	1,486 (6.2%)	1,431 (6.0%)	1,357 (5.7%)	1,369 (5.8%)	1,277 (5.4%)
Intake	234 [0.1–378]	525 [378–664]	791 [664–913]	1,046 [913–1,196]	1,398 [1,196–3,611]	6.4 [1.8–7.3]	8.0 [7.3–8.7]	9.4 [8.7–10.2]	11.0 [10.2–12.0]	13.2 [12.0–19.0]
HR (95% CI)										
Model 1 (minimally adjusted)	Ref.	**0.83 (0.77, 0.90)**	**0.81 (0.75, 0.87)**	**0.76 (0.71, 0.83)**	**0.75 (0.69, 0.81)**	Ref.	**0.92 (0.85, 0.99)**	**0.84 (0.77, 0.90)**	**0.82 (0.76, 0.88)**	**0.76 (0.70, 0.83)**
Model 2 (+ demographics)	Ref.	**0.86 (0.80, 0.93)**	**0.85 (0.79, 0.92)**	**0.81 (0.75, 0.88)**	**0.80 (0.74, 0.87)**	Ref.	0.95 (0.88, 1.02)	**0.88 (0.81, 0.95)**	**0.87 (0.80, 0.94)**	**0.82 (0.76, 0.90)**
Model 3 (+ lifestyle)	Ref.	**0.88 (0.81, 0.95)**	**0.88 (0.82, 0.95)**	**0.84 (0.78, 0.91)**	**0.83 (0.76, 0.90)**	Ref.	0.98 (0.91, 1.05)	**0.91 (0.85, 0.99)**	**0.91 (0.84, 0.99)**	**0.87 (0.80, 0.94)**
Model 4 (+ diet)	Ref.	**0.90 (0.83, 0.97)**	**0.92 (0.85, 0.99)**	**0.89 (0.82, 0.96)**	**0.88 (0.81, 0.96)**	Ref.	0.99 (0.92, 1.07)	0.93 (0.86, 1.01)	0.93 (0.86, 1.01)	**0.89 (0.82, 0.98)**
Model 5 (+ medical history)	Ref.	**0.91 (0.84, 0.98)**	0.92 (0.85, 1.00)	**0.89 (0.82, 0.97)**	**0.88 (0.81, 0.96)**	Ref.	0.99 (0.92, 1.07)	0.94 (0.87, 1.01)	0.94 (0.86, 1.02)	**0.90 (0.82, 0.98)**
**T2DM incidence**										
Population	23,921	23,920	23,920	23,920	23,920	23,921	23,920	23,920	23,920	23,920
No. of events	847 (3.5%)	667 (2.8%)	650 (2.7%)	629 (2.6%)	628 (2.6%)	832 (3.5%)	690 (2.9%)	686 (2.9%)	649 (2.7%)	564 (2.4%)
Intake	236 [0.1–382]	529 [382–668]	795 [668–917]	1,049 [917–1,198]	1,400 [1,198–3,611]	6.4 [1.8–7.3]	8.0 [7.3–8.7]	9.4 [8.7–10.2]	11.0 [10.2–12.0]	13.2 [12.0–19.0]
HR (95% CI)										
Model 1 (minimally adjusted)	Ref.	**0.72 (0.65, 0.79)**	**0.60 (0.54, 0.67)**	**0.53 (0.47, 0.59)**	**0.48 (0.43, 0.54)**	Ref.	**0.76 (0.69, 0.85)**	**0.70 (0.63, 0.77)**	**0.61 (0.54, 0.68)**	**0.51 (0.45, 0.57)**
Model 2 (+ demographics)	Ref.	**0.82 (0.74, 0.91)**	**0.74 (0.67, 0.83)**	**0.68 (0.61, 0.76)**	**0.65 (0.58, 0.73)**	Ref.	**0.86 (0.77, 0.95)**	**0.82 (0.74, 0.91)**	**0.75 (0.67, 0.83)**	**0.65 (0.58, 0.73)**
Model 3 (+ lifestyle)	Ref.	**0.86 (0.78, 0.95)**	**0.80 (0.72, 0.89)**	**0.75 (0.67, 0.84)**	**0.70 (0.62, 0.78)**	Ref.	0.91 (0.82, 1.01)	0.90 (0.80, 1.00)	**0.84 (0.75, 0.94)**	**0.76 (0.67, 0.86)**
Model 4 (+ diet)	Ref.	**0.88 (0.79, 0.97)**	**0.83 (0.74, 0.92)**	**0.79 (0.70, 0.88)**	**0.74 (0.66, 0.84)**	Ref.	0.92 (0.83, 1.02)	0.92 (0.83, 1.03)	**0.87 (0.78, 0.98)**	**0.80 (0.70, 0.90)**
Model 5 (+ medical history)	Ref.	**0.88 (0.79, 0.98)**	**0.83 (0.75, 0.93)**	**0.79 (0.70, 0.89)**	**0.75 (0.66, 0.84)**	Ref.	0.93 (0.84, 1.03)	0.93 (0.83, 1.04)	**0.88 (0.78, 0.99)**	**0.80 (0.70, 0.91)**
**Cancer incidence**										
Population	22,444	22,443	22,444	22,443	22,444	22,444	22,443	22,444	22,443	22,444
No. of events	1,804 (8.0%)	1,844 (8.2%)	1,881 (8.4%)	1,993 (8.9%)	1,919 (8.6%)	1,929 (8.6%)	1,870 (8.3%)	1,814 (8.1%)	1,946 (8.7%)	1,882 (8.4%)
Intake	233 [0.1–376]	522 [376–662]	789 [662–912]	1,045 [912–1,195]	1,398 [1,195–3,611]	6.4 [1.8–7.3]	8.0 [7.3–8.7]	9.4 [8.7–10.2]	11.0 [10.2–11.9]	13.1 [11.9–19.0]
HR (95% CI)										
Model 1 (minimally adjusted)	Ref.	0.94 (0.88, 1.00)	**0.92 (0.86, 0.98)**	0.95 (0.89, 1.01)	**0.90 (0.84, 0.96)**	Ref.	**0.93 (0.87, 0.99)**	**0.89 (0.83, 0.95)**	0.94 (0.87, 1.00)	**0.90 (0.84, 0.96)**
Model 2 (+ demographics)	Ref.	0.95 (0.89, 1.02)	0.94 (0.88, 1.00)	0.97 (0.90, 1.03)	**0.92 (0.85, 0.98)**	Ref.	0.94 (0.88, 1.00)	**0.90 (0.84, 0.96)**	0.95 (0.89, 1.02)	**0.91 (0.85, 0.98)**
Model 3 (+ lifestyle)	Ref.	0.96 (0.90, 1.03)	0.95 (0.89, 1.02)	0.99 (0.92, 1.05)	0.93 (0.87, 1.00)	Ref.	0.95 (0.89, 1.01)	**0.92 (0.86, 0.98)**	0.97 (0.90, 1.04)	**0.92 (0.86, 0.99)**
Model 4 (+ diet)	Ref.	0.96 (0.90, 1.03)	0.94 (0.88, 1.01)	0.97 (0.91, 1.04)	**0.91 (0.85, 0.99)**	Ref.	0.95 (0.89, 1.01)	**0.91 (0.85, 0.97)**	0.96 (0.90, 1.03)	**0.92 (0.85, 0.99)**
Model 5 (+ medical history)	Ref.	0.96 (0.90, 1.03)	0.94 (0.88, 1.01)	0.97 (0.91, 1.05)	**0.92 (0.85, 0.99)**	Ref.	0.95 (0.89, 1.01)	**0.91 (0.85, 0.98)**	0.96 (0.90, 1.03)	**0.92 (0.85, 0.99)**
**Respiratory disease incidence**										
Population	20,887	20,887	20,886	20,887	20,887	20,887	20,887	20,886	20,887	20,887
No. of events	2,754 (13.2%)	2,440 (11.7%)	2,491 (11.9%)	2,533 (12.1%)	2,727 (13.1%)	2,836 (13.6%)	2,553 (12.2%)	2,477 (11.9%)	2,572 (12.3%)	2,507 (12.0%)
Intake	237 [0.1–382]	531 [382–669]	795 [669–917]	1,049 [917–1,199]	1,400 [1,199–3,611]	6.4 [2.0–7.3]	8.0 [7.3–8.7]	9.4 [8.7–10.2]	11.0 [10.2–11.9]	13.1 [11.9–19.0]
HR (95% CI)										
Model 1 (minimally adjusted)	Ref.	**0.81 (0.76, 0.85)**	**0.78 (0.74, 0.82)**	**0.75 (0.71, 0.79)**	**0.78 (0.74, 0.83)**	Ref.	**0.86 (0.81, 0.90)**	**0.81 (0.76, 0.85)**	**0.81 (0.76, 0.86)**	**0.78 (0.74, 0.83)**
Model 2 (+ demographics)	Ref.	**0.84 (0.79, 0.88)**	**0.82 (0.78, 0.87)**	**0.80 (0.75, 0.85)**	**0.84 (0.79, 0.89)**	Ref.	**0.89 (0.84, 0.94)**	**0.85 (0.80, 0.89)**	**0.86 (0.81, 0.91)**	**0.84 (0.79, 0.89)**
Model 3 (+ lifestyle)	Ref.	**0.86 (0.81, 0.91)**	**0.86 (0.81, 0.91)**	**0.84 (0.79, 0.89)**	**0.88 (0.83, 0.93)**	Ref.	**0.92 (0.87, 0.97)**	**0.89 (0.84, 0.94)**	**0.91 (0.86, 0.97)**	**0.89 (0.84, 0.95)**
Model 4 (+ diet)	Ref.	**0.87 (0.82, 0.92)**	**0.88 (0.83, 0.93)**	**0.86 (0.81, 0.92)**	**0.91 (0.85, 0.96)**	Ref.	**0.93 (0.88, 0.99)**	**0.91 (0.85, 0.96)**	**0.93 (0.87, 0.99)**	**0.92 (0.86, 0.98)**
Model 5 (+ medical history)	Ref.	**0.87 (0.83, 0.92)**	**0.89 (0.84, 0.94)**	**0.87 (0.82, 0.92)**	**0.91 (0.86, 0.97)**	Ref.	**0.93 (0.88, 0.99)**	**0.91 (0.86, 0.96)**	**0.93 (0.88, 0.99)**	**0.92 (0.86, 0.98)**
**Neurodegenerative disease incidence**										
Population	24,902	24,902	24,902	24,902	24,902	24,902	24,902	24,902	24,902	24,902
No. of events	397 (1.6%)	355 (1.4%)	404 (1.6%)	401 (1.6%)	364 (1.5%)	327 (1.3%)	381 (1.5%)	414 (1.7%)	412 (1.7%)	387 (1.6%)
Intake	234 [0.1–378]	526 [378–665]	792 [665–915]	1,048 [915–1,197]	1,400 [1,197–3,611]	6.4 [1.8–7.3]	8.0 [7.3–8.7]	9.4 [8.7–10.2]	11.0 [10.2–11.9]	13.1 [11.9–19.0]
HR (95% CI)										
Model 1 (minimally adjusted)	Ref.	**0.77 (0.66, 0.88)**	**0.81 (0.71, 0.94)**	**0.78 (0.67, 0.90)**	**0.71 (0.61, 0.83)**	Ref.	1.06 (0.91, 1.23)	1.10 (0.94, 1.27)	1.04 (0.90, 1.22)	0.98 (0.83, 1.15)
Model 2 (+ demographics)	Ref.	**0.78 (0.68, 0.91)**	**0.84 (0.73, 0.96)**	**0.80 (0.69, 0.93)**	**0.73 (0.63, 0.86)**	Ref.	1.08 (0.93, 1.25)	1.12 (0.97, 1.31)	1.08 (0.92, 1.26)	1.01 (0.86, 1.19)
Model 3 (+ lifestyle)	Ref.	**0.79 (0.68, 0.91)**	**0.85 (0.74, 0.98)**	**0.82 (0.71, 0.95)**	**0.75 (0.64, 0.88)**	Ref.	1.11 (0.96, 1.30)	1.16 (1.00, 1.35)	1.10 (0.94, 1.29)	1.06 (0.89, 1.25)
Model 4 (+ diet)	Ref.	**0.81 (0.70, 0.93)**	0.88 (0.76, 1.02)	0.86 (0.73, 1.00)	**0.79 (0.67, 0.93)**	Ref.	1.11 (0.96, 1.30)	1.16 (1.00, 1.36)	1.10 (0.93, 1.30)	1.06 (0.89, 1.27)
Model 5 (+ medical history)	Ref.	**0.82 (0.71, 0.95)**	0.90 (0.77, 1.04)	0.87 (0.75, 1.01)	**0.80 (0.68, 0.94)**	Ref.	1.11 (0.96, 1.30)	1.17 (1.00, 1.37)	1.11 (0.94, 1.31)	1.07 (0.90, 1.28)

HRs (95% CI) for risk of all-cause mortality and major chronic diseases obtained from Cox proportional-hazards models with age as the underlying timescale. HRs and 95% CIs in bold do not include 1.00. All models considering diversity of flavonoid intake were adjusted for quantity of flavonoid intake and vice versa. Model 1, adjusted for: sex, region of residence and number of dietary recalls. Model 2, multivariable adjusted for model 1 plus: ethnicity, BMI, education and socioeconomic status (Townsend deprivation index). Model 3, multivariable adjusted for model 2 plus: smoking, physical activity and alcohol intake. Model 4, multivariable adjusted for model 3 plus: intakes (g d^−1^) of red and processed meat, refined grains, whole grains, sugary drinks, coffee, saturated fatty acids, sodium and energy (kcal d^−1^). Model 5, multivariable adjusted for model 4 plus history of diabetes (type 1 or 2; not adjusted in T2DM analysis), hypertension and hypercholesterolaemia and for analysis of all-cause mortality, further adjusted for prevalent CVD, cancer, respiratory disease and neurodegenerative disease at baseline. Sample sizes differ between analyses due to differing per-analysis exclusions. Intake shows flavonoid quantity (mg d^−1^) and diversity (Hill number per day) as median [min–max]; intake quintiles are mutually exclusive. Ref., reference.

### Flavonoid subclasses, all-cause mortality and chronic disease

Minimally (model 1) and multivariable adjusted models (models 2–5) for diversity of individual flavonoid subclasses and the risk of all-cause mortality and major chronic disease are presented in Supplementary Table 5. Overall, following adjustment for demographic and lifestyle factors (model 3), further adjustments for diet (model 4) and medical history (model 5) did not substantially alter the findings. In the fully adjusted model (model 5), the wider diversities of intake of compounds within the flavan-3-ol and flavanone subclasses were each associated with a lower risk of all-cause mortality, independent of absolute intake; the HR remained stable after both Q4 and Q2 respectively (HR (95% CI) for flavan-3-ols Q4 versus Q1, 0.91 (0.83, 0.99); flavanones Q2 versus Q1, 0.90 (0.83, 0.98); model 5; Table 3 and Supplementary Table 5). When the corresponding model terms for quantity of consumption were examined, only flavan-3-ol intake was associated with lower risk of all-cause mortality; the HRs were relatively stable beyond Q2 (Q2 versus Q1, 0.85 (0.78, 0.93); Supplementary Table 6). The data for chronic disease outcomes reveal that, compared with lower intakes (Q1), significant associations mostly emerged in those with the widest diversity at and above Q4; for flavan-3-ols there was a 13% (Q5, 0.87 (0.77, 0.98)) and an 8% (Q4, 0.92 (0.86, 0.99)) lower risk of T2DM and cancer; for flavanone there was a 7% (Q5, 0.93 (0.88, 0.99)) and a 6% (Q5, 0.93 (0.87, 0.99)) lower risk of cancer and respiratory disease; and for flavones there was a 13% (Q4, 0.89 (0.80, 0.99)) and an 18% (Q5, 0.82 (0.71, 0.95)) lower risk of T2DM and neurodegenerative disease, respectively (model 5; Table 3 and Supplementary Table 5). When we examined the models for the subclasses showing beneficial associations for diversity, associations for quantity of intake emerged with T2DM wherein participants at and above Q3 for flavones and Q4 for flavan-3-ols were at a lower risk (flavones Q3, 0.89 (0.80, 0.99); flavan-3-ols Q4, 0.85 (0.77, 0.95); model 5; Supplementary Table 6). No interactions were observed between quantity and diversity of intake of any subclass with any outcome (*P*_interaction_ all >0.05 (model 5)).

**Table 3:** Diversity of intake of flavonoid-rich foods and individual flavonoid subclasses associate with risk of all-cause mortality and incidence of major chronic disease

	Diversity of intake of:											
	Flavonoid-rich foods		Flavan-3-ols		Anthocyanins		Flavonols		Flavanones		Flavones	
	Q1	Q5	Q1	Q5	Q1	Q5	Q1	Q5	Q1	Q5	Q1	Q5
All-cause mortality	Ref.	**0.84 (0.76, 0.91)**	Ref.	**0.90 (0.82, 0.99)**	Ref.	1.02 (0.93, 1.11)	Ref.	0.98 (0.89, 1.07)	Ref.	**0.88 (0.81, 0.96)**	Ref.	0.93 (0.85, 1.01)
CVD	Ref.	0.94 (0.86, 1.02)	Ref.	0.94 (0.86, 1.02)	Ref.	1.00 (0.92, 1.10)	Ref.	1.04 (0.96, 1.13)	Ref.	1.00 (0.93, 1.08)	Ref.	0.94 (0.87, 1.01)
T2DM	Ref.	0.94 (0.83, 1.06)	Ref.	**0.87 (0.77, 0.98)**	Ref.	0.92 (0.82, 1.04)	Ref.	1.09 (0.97, 1.23)	Ref.	0.91 (0.82, 1.01)	Ref.	**0.87 (0.78, 0.97)**
Cancer incidence	Ref.	0.93 (0.87, 1.00)	Ref.	**0.92 (0.86, 0.99)**	Ref.	1.01 (0.94, 1.09)	Ref.	0.98 (0.91, 1.05)	Ref.	**0.93 (0.87, 0.99)**	Ref.	0.96 (0.90, 1.02)
Respiratory disease	Ref.	**0.92 (0.87, 0.98)**	Ref.	0.97 (0.91, 1.03)	Ref.	0.95 (0.89, 1.01)	Ref.	0.98 (0.92, 1.04)	Ref.	**0.94 (0.89, 0.99)**	Ref.	0.97 (0.92, 1.03)
Neurodegenerative disease	Ref.	0.99 (0.84, 1.15)	Ref.	1.08 (0.92, 1.27)	Ref.	0.92 (0.78, 1.09)	Ref.	0.93 (0.79, 1.09)	Ref.	1.15 (0.99, 1.34)	Ref.	**0.82 (0.71, 0.95)**

HRs (95% CI) for risk of all-cause mortality and major chronic diseases obtained from Cox proportional-hazards models with age as the underlying timescale. HR and 95% CIs in bold do not include 1.00. All models considering diversity of servings of flavonoid-rich food intake were adjusted for quantity of servings of the same flavonoid-rich foods. All models considering flavonoid subclasses were additionally individually adjusted for quantity of intake of the specific flavonoid subclass of interest. All models were adjusted for sex, region of residence, number of dietary recalls, ethnicity, BMI, education, socioeconomic status (Townsend deprivation index), smoking, physical activity, alcohol intake, plus intakes (g d^−1^) of red and processed meat, refined grains, whole grains, sugary drinks, coffee, saturated fatty acids, sodium and energy (kcal d^−1^), and for history of diabetes (type 1 or 2; not adjusted in T2DM analysis), hypertension and hypercholesterolaemia; for analysis of all-cause mortality, models were further adjusted for prevalent CVD, cancer, respiratory disease and neurodegenerative disease at baseline. Further model details (including Q2–Q3, case events and sample sizes) can be found in Supplementary Table 5. Flavan-3-ols includes monomers, proanthocyanidins and theaflavins/thearubigins.

### Flavonoid-rich foods, all-cause mortality and chronic disease

Minimally (model 1) and multivariable adjusted models (models 2–5) for diversity of flavonoid-rich foods are presented in Supplementary Table 5. Adjustment beyond demographic and lifestyle factors (model 3) for participant diet (model 4) and medical history (model 5) did not appreciably affect the associations. In the fully adjusted model, when holding the quantity of intake constant, the risk of all-cause mortality was progressively lower among those with a higher diversity of flavonoid-rich food intake; compared with an effective serving of 1.3, those with 2, 2.7, 3.4 and 4.5 different effective servings were associated with an 8% (0.92 (0.85, 1.00)), 10% (0.91 (0.84, 0.99)), 13% (0.88 (0.81, 0.96)) and 16% (0.84 (0.76, 0.91)) lower risk of all-cause mortality, respectively (model 5; Table 3 and Supplementary Table 5). Holding the diversity of intake constant, there was no clear association for consuming a higher quantity of flavonoid-rich foods (model 5; Supplementary Table 6). Examination of chronic disease outcomes revealed that those with the highest (versus lowest) diversity of flavonoid-rich food intake had an 8% lower risk of respiratory disease (0.92 (0.87, 0.98)); there were no compelling associations with other endpoints (model 5; Table 3 and Supplementary Table 5). Holding diversity constant, a higher quantity of flavonoid-rich foods, beyond Q2 (Q2, 0.87 (0.78, 0.97)), associated with a lower risk of T2DM; there were no compelling associations with other endpoints (model 5; Supplementary Table 6). No interactions (*P*_interaction_ all >0.05 (Model 5)) were observed between quantity and diversity of flavonoid-rich food consumption.

### Sensitivity analyses

Neither removing energy intake nor adjusting for a healthy plant-based diet score substantively altered the HR (sensitivity analyses 1 and 2; Supplementary Tables 5 and 7). Excluding participants who had an event in the first two years of follow-up tended to marginally strengthen the relationships between our exposures and outcomes (sensitivity analysis 3; Supplementary Tables 5 and 7).

## Discussion

In >120,000 UK Biobank participants, we observed that participants who consumed the widest diversity of dietary flavonoids, flavonoid-rich foods and/or specific flavonoid subclasses had a lower risk of all-cause mortality and incidence of cause-specific chronic disease, ranging from cardiometabolic disorders (including CVD and T2DM) to other major conditions, such as cancer, respiratory disease and neurodegenerative disease. We also found that both the quantity and diversity of total dietary flavonoids are independent predictors of mortality and several chronic diseases, suggesting that consuming a higher quantity and wider diversity is better for longer-term health than higher intakes of either component alone.

Our findings highlight the importance of consuming a diverse range of flavonoids for the management of chronic disease risk, which, from a public health perspective, provides support for consuming a variety of flavonoid-rich foods such as green and/or black tea, berries, apples, oranges and grapes^25^. This fits with our current understanding that different flavonoid compounds can exert different biological benefits^1,26–28^. For example, in the regulation of blood pressure alone, compounds from each subclass appear to act on a variety of different mechanisms, increasing nitric oxide bioavailability, reducing endothelial cell oxidative stress and modulating vascular ion channel activity^29,30^. Indeed, the health-promoting effects of flavonoids are wide ranging, with multiple flavonoid compounds implicated in multiple biological activities, including, among others, inhibiting platelet aggregation, lowering low-density lipoprotein oxidation, mitigating atherosclerotic lesion formation, improving insulin sensitivity indices, inducing antioxidant defences, and reducing inflammatory responses in addition to specific anticarcinogenic actions, such as an ability to induce apoptosis in tumour cells, inhibit cancer cell proliferation, and prevent angiogenesis and tumour cell invasion^15,28^. As a result, the collective actions of multiple flavonoids appear to lead to greater health protection compared with single subclasses or compounds.

We found that consuming both a higher quantity and wider diversity of dietary flavonoids appears better for longer-term health than higher intakes of either component alone. To date, epidemiological research has focused on the quantity of flavonoid intake, finding that higher consumption of several flavonoid subclasses is associated with a lower risk of several chronic diseases^2,7–12,31,32^. Indeed, the first proposed dietary guideline for flavonoids was released in 2022^33^, and recommended consumption of 400–600 mg d^−1^ of flavan-3-ols for potential cardiometabolic health benefits. Our results suggest that future guidelines could be reframed to also consider recommending intake from a range of sources. Further studies are also ongoing to determine the environmental footprints of different flavonoid-rich foods to ensure their consumption also supports environmental sustainability and planetary health^34^. Moreover, our findings also align with our other recent work in which we propose a composite measure of flavonoid intake (termed the Flavodiet score) which is a sum of servings of flavonoid-rich foods^6^. We observed that those who had a better Flavodiet score had a lower risk of all-cause mortality^6^. Our current study on flavonoid diversity and health outcomes supports the Flavodiet score concept as means to promote higher intakes of flavonoids from different sources. Our analysis of diversity also complements existing analyses that evaluate associations between specific flavonoid food sources and health outcomes, which enhance the evidence base for the health benefits of specific flavonoid-rich foods^35,36^. However, by studying diversity specifically, our results suggest that consuming a greater variety of such sources appears better than their intakes in isolation.

To estimate flavonoid diversity, we used Shannon’s equation with Hill’s numbers^22–24^. This provides an approach to explicitly separate out and study the independent benefits of flavonoid diversity, versus quantity, for health outcomes. A fundamental feature of the Shannon equation is that it considers the most diverse diets to consist of all flavonoids consumed in equal proportions. Although this reflects a technical definition of diversity, such an intake is unlikely to occur in the real world and may not be the pattern of consumption that offers the greatest health benefits. Shannon’s equation also only permits calculation of diversity among flavonoid consumers (omitting non-consumers), and results should be interpreted within this context (although <0.01% of participants in this cohort did not consume any flavonoids). We must also consider that calculating diversity within individual subclasses does not account for diversity of other subclasses (which appears important) and that calculating diversity by way of major flavonoid-rich foods does not account for other flavonoid sources (which may potentially be major sources for some individuals). While calculating flavonoid diversity by way of total flavonoid compound intake appears to overcome these limitations, this method relies on the precision of compound intake estimates, and these estimates, given the inherent limitations of dietary assessment methods and nutrient composition databases^37^, are likely to be relatively crude. Nevertheless, even with these constraints, we observed a significantly lower risk of all-cause mortality and cause-specific chronic disease among those with the most (compared with the least) diverse flavonoid intakes when using this method. Indeed, beyond flavonoids this method could be further used to estimate and evaluate diversity of other (poly)phenolics, or groups of bioactives, or potentially various food groups. Although there have been recent discussion and some previous use of various diversity indices in nutrition science^38–41^, Shannon’s equation (with Hill numbers) does not seem to have been used before to partition and study the independent roles of diversity and quantity. Hence, this work introduces a potential approach to study these characteristics of other dietary components in the future.

No previous works appear to have reported on the human health benefits of a flavonoid-diverse diet. Consequently, replication of our findings in other cohorts and clinical trials will be critical, as will the exploration of flavonoid diversity with other disease outcomes. Interpretation, however, requires careful consideration. For the most part, we observed that both quantity and diversity were independent predictors, suggesting there is a benefit to consuming a higher diversity beyond that of simply consuming a high quantity (and vice versa), although this relationship did not interact such that the benefit together was even greater than the combination of the individual parts^42^. On other occasions we observed quantity but not diversity was a predictor, which could suggest consuming a higher amount of any type provides benefit. Or perhaps a wider diversity of intake within the population under study is required before a role for diversity becomes observable, or that the average compositional make-up of diversity within the population was not relevant to the disease in question. Certainly, the biological relevance of diversity within subclasses may be less important if at least some compounds have similar biological effects. Indeed, those with the lowest diversity could theoretically consume one flavonoid type alone; hypothetically speaking, if this was considered the reference group and compared to those with a wider diversity, then after adjustment for quantity, the comparison compares one against multiple different flavonoid types, holding total quantity constant. If the one flavonoid type was overly protective against the disease in question, then there may be no benefit to consuming a wider diversity if the other flavonoids do not collectively provide a benefit larger than the reference. In other analyses we observed that only flavonoid diversity, but not quantity, predicted the outcomes. This could be due to synergies between different flavonoids, whereas simply consuming higher amounts of less diverse compounds may afford no benefit. We also observed that the quantity and diversity of flavonoid compound intake but not servings of flavonoid-rich foods were significantly associated with more outcomes, suggesting that the absolute intake of flavonoids matters more than the servings of flavonoid-rich foods per se, potentially because different foods have varying flavonoid densities and serving sizes. Moreover, combinations of some foods will probably provide a greater diversity of flavonoids than others—or example, consuming red wine and grapes will probably be less diverse than consuming oranges and grapes because there is less overlap in the flavonoid profiles of the foods.

The strengths of this study include the prospective design, large sample size, high number of cases and long follow-up time of ~10 years. Several limitations, however, should be noted. First, the observational design restricts our ability to infer causality or to exclude the possibility of residual confounding. To this end, we must consider whether the associations observed represent a benefit of higher diversity of flavonoids per se, or a signal that the various flavonoids act synergistically with other compounds found in flavonoid-rich foods, such as phenolic acids, lignans or other bioactives^2^. Indeed, the possibility of flavonoids being a marker of other unobserved and potential protective factors cannot be discounted. Second, although the Oxford WebQ has been validated against biomarkers and 24-h recalls for selected nutrients^43,44^, it does not capture data on certain types of flavonoid-rich foods (for example, specific types of berries), which potentially leads to imprecision in the assessment of diversity for certain subclasses (for example, anthocyanins), and as with all self-reported dietary assessments, common limitations and reporting biases apply^2,45^. Moreover, due to the limited number of dietary assessments, our analyses may have been affected by regression dilution with a probable underestimation of the strengths of associations^46^; this may be of specific importance when assessing diversity, assuming variation in intake is greater over longer timeframes. Third, incidence of T2DM was ascertained based on hospital and death records, which may not capture all cases, such as those diagnosed and treated in primary care. This may have introduced some degree of error, particularly if hospitalized individuals have different health-seeking behaviours or characteristics than those treated in primary care, highlighting the need for additional studies. Fourth, potential confounders were only assessed at baseline, and it is unclear how potential changes in their trajectories may have impacted upon the observed associations. Fifth, although we conducted extensive analysis showing that the associations of our exposures with the outcomes appear robust, we acknowledge that multiplicity issues should be considered when interpreting the results. Sixth, given our sample is not representative of all populations in terms of age, ethnicity, health status or socioeconomic standing, and so on, the generalizability of our results requires confirmation in other populations.

In conclusion, we found that a wider diversity of intake of total flavonoids, flavonoid-rich foods and/or specific flavonoid subclasses is associated with a lower risk of all-cause mortality and incidence of chronic disease, including CVD, T2DM, cancer, respiratory disease and neurodegenerative disease. We also observed that a higher quantity and wider diversity of dietary flavonoids, when consumed together, may represent the optimal approach for improving long-term health, compared with increasing either flavonoid quantity or diversity alone. Overall, our findings suggest simple and achievable dietary changes such as including several different daily servings of flavonoid-rich foods or beverages, such as tea, berries, apples, oranges or grapes, might have a major impact on population health, lowering the risk of all-cause mortality and major chronic disease.

## Methods

### Design

For the present investigation, we used data from the UK Biobank—a large, prospective, population-based cohort study^47^. Between 2006 and 2010, >500,000 male and female adults, aged 40–69 yr, were enrolled^47^. Participants attended one of 22 assessment centres located across England, Scotland and Wales, where they undertook a comprehensive baseline assessment, completing questionnaires and physical measures, and provided biological samples. The UK Biobank study received ethical approval from the NHS North West Multi-Centre Research Ethics Committee (reference 11/NW/0382) and all participants provided informed consent.

For the current analysis, we excluded participants who withdrew their consent during follow-up or who completed fewer than two 24-h dietary questionnaires (by first removing individual recalls without plausible energy intakes: <800 or >4,200 kcal d^−1^ for men and <500 or >3,500 kcal d^−1^ for women) (Supplementary Fig. 1). Additionally, for the respective outcomes of interest, we excluded participants with prevalent CVD, T2DM, cancer, respiratory disease or neurodegenerative disease, prior to the last date of dietary assessment (Supplementary Table 8). Lastly, because Shannon’s equation requires intake of at least one kind of flavonoid compound, those with zero total flavonoid intake were excluded, and then, depending on the exposure of interest (flavonoid-rich foods or intra-subclass diversity, and so on), participants with zero intake of flavonoid-rich foods or specific subclasses were excluded on a per-analysis basis, because the collective exclusion at the flavonoid-rich food or intra-subclass level would bias diversity of other levels (for example, compounds (Supplementary Fig. 1)).

### Exposures

Dietary information was collected using the Oxford WebQ 24-h dietary questionnaire^44^, which participants completed on up to five separate occasions, between 2009 and 2012^48^. Flavonoid intake was estimated from the Oxford WebQ 24-h dietary questionnaire using the US Department of Agriculture flavonoid and proanthocyanidin food content databases^49,50^, with food codes derived from the updated version of the nutrient calculations for the Oxford WebQ for food items and composite recipes^13,51^. Flavonoid intakes (mg d^−1^) from all completed questionnaires with plausible energy intakes were averaged. We derived intakes of several flavonoids subclasses as follows: flavonols (quercetin, kaempferol, myricetin and isorhamnetin), anthocyanins (cyanidin, delphinidin, malvidin, pelargonidin, petunidin and peonidin), flavan-3-ols ((+)-catechin, (+)-gallocatechin, (−)-epicatechin, (−)-epigallocatechin, (−)-epicatechin 3-gallate and (−)-epigallocatechin-3-gallate, plus dimers, trimers, 4–6-mers, 7–10-mers and polymers, plus theaflavin, theaflavin-3-gallate, theaflavin-3′-gallate, theaflavin-3,3′-digallate and thearubigins), flavanones (eriodictyol, hesperetin and naringenin) and flavones (luteolin and apigenin). Total flavonoid intake was calculated as the sum of all compounds. Intakes of isoflavones were not calculated due to the low consumption of isoflavone-containing foods in the general UK population^52^.

Diversity of flavonoid intake was calculated using Shannon’s equation for entropy^22^ which was subsequently converted to Hill’s effective numbers^23,24^. Calculations of diversity were made for total flavonoid intake, which considered diversity of all 31 flavonoids as described above. In an exploratory analysis we examined (1) intra-subclass diversity, which considered diversity of intake within individual subclasses, and (2) servings of flavonoid-rich foods, which included the key contributors to each flavonoid subclass, including tea (black and green), red wine, apples, berries, grapes, oranges (including satsumas), grapefruit, sweet peppers, onions and dark chocolate. The key contributors were determined as the three foods that contributed the highest percentage to the intakes of each flavonoid subclass (excluding fruit juices), and dark chocolate was included as it is typically high in flavan-3-ols^13^. Shannon’s equation is as follows:

$${\rm{Shannon}}\; {\rm{index}}\left({H}\;\right)=-\mathop{\sum }\limits_{i=1}^{s}{p}_{i}\mathrm{ln}\,{p}_{i}$$

In Shannon’s equation, *p*_*i*_ is calculated as the proportion of individual flavonoids consumed per day (that is, the quantity of compounds (mg d^−1^) or flavonoid-rich foods (servings per day)) relative to total intake (that is, the total quantity of flavonoids (mg d^−1^) or flavonoid-rich foods (servings per day)) and *s* is the total number of individual flavonoid types (that is, compounds or flavonoid-rich foods) consumed. Diversity of flavonoid intake was calculated using the R package Vegan^53^. Conversion of Shannon’s score into Hill’s effective numbers was undertaken by exponentiating *H* (refs. ^23,24^).

The purpose of using effective numbers is to convert Shannon’s non-linear score into an interpretable metric that quantifies diversity^23^. The resulting output, termed effective numbers, shows the number of different types of flavonoids that would need to be consumed in a specific proportional make-up to meet the same relative diversity as the diet from which it was calculated, wherein a higher value indicates wider diversity (a detailed explanation of effective numbers can be found in the Supplementary Methods). The Shannon equation and Hill numbers produce a measure of diversity that is relative to, and independent of, the quantity of flavonoid intake, such that it is possible that two individuals can have exactly the same diversity score, yet one of them may consume, for example, a threefold higher quantity of flavonoids. Therefore, following statistical adjustment for quantity of flavonoid consumption, it is possible to study the independent benefit of diversity of flavonoid intake.

### Outcomes

The outcomes in the current study were all-cause mortality and incidence (first-time fatal or non-fatal events) of CVD, T2DM, total cancer, respiratory disease, and neurodegenerative disease. Date of death was obtained from death certificates held by the National Health Service Information Centre (England and Wales) and the National Health Service Central Register Scotland (Scotland). Dates and causes of hospital admissions were identified via record linkage to Health Episode Statistics (England), the Patient Episode Database (Wales) and the Scottish Morbidity Records (Scotland) as well as the National Cancer Registries (England, Scotland and Wales). Incident outcomes were defined as a hospital admission or death identified through primary or secondary diagnosis codes using International Classification of Diseases, Tenth Revision (ICD-10) as follows: CVD (I20-I25, I63 and I70-I74), T2DM (E11), cancer (C00-C97, excluding non-melanoma skin cancer (C44)), respiratory disease (J09-J98, I26 and I27) and neurodegenerative disease (F00–03, G12.2, G20, G21, G23.1–23.3, G23.8, G23.9, G30 and G31). Hospital admission follow-up data for CVD, T2DM, respiratory disease and neurodegenerative disease were available until 31 October 2022 for England, 31 August 2022 for Scotland and 31 May 2022 for Wales. Follow-up data for cancer were available until 31 December 2016 for Wales, 31 December 2020 for England and 30 November 2021 for Scotland. Mortality data were available until 30 November 2022 for England, Scotland and Wales. We therefore censored outcome analyses on these dates.

### Covariates

Information on demographics, lifestyle factors and medical history including sex, age, ethnicity, anthropometry, physical activity, education, smoking and alcohol habits were obtained from the baseline assessment. Anthropometric measurements (height and weight) were obtained by trained personnel. BMI was calculated as weight/(height^2^) (kg m^−^^2^). Physical activity was derived using the International Physical Activity Questionnaire short form, and total physical activity was calculated as the sum of walking, moderate and vigorous activity measured as metabolic equivalents (MET-h per week). Area-based socioeconomic status was derived from postal code of residence using the Townsend deprivation score. History of hypertension and diabetes mellitus (type 1 or 2) was derived from self-reported physician diagnosis of disease or medication use at recruitment, and from ICD codes dated prior to the last date of dietary assessment (Supplementary Table 8). History of hypercholesterolaemia was identified by physician diagnosis (self-reported) or the taking of cholesterol-lowering medication (Supplementary Table 8). To identify other baseline comorbidities, self-reported physician-diagnosed CVD, cancer, neurodegenerative disease and respiratory disease at recruitment was combined with ICD codes dated prior to the last date of diet assessment (Supplementary Table 8). The Oxford WebQ was used to calculate average daily intakes of foods, nutrients, energy intake via information recorded in the UK Nutrient Databank as previously reported^54^. The healthful plant-based diet index was derived from 17 food groups^55^.

### Statistical analysis

Cox proportional-hazards models were used to investigate relationships between diversity of flavonoid consumption and all outcomes of interest. Participants were followed up from the completion of the last valid diet questionnaire until the first occurrence of the outcome event, death, loss to follow-up or the end of follow-up (as described above), whichever occurred first. Flavonoid diversity was modelled as quintiles with low flavonoid diversity (Q1) as the reference group. All models examining diversity were mutually adjusted for quantity (quintiles) of the same flavonoids that contributed to flavonoid diversity. All models used age as the underlying timescale^56^. Five models of adjustment were computed: model 1 minimally adjusted for sex, region of residence (entered as a strata variable: London, North West England, North East England, Yorkshire, West Midlands, East Midlands, South East England, South West England, Scotland and Wales) and number of dietary assessments completed with plausible energy intake (2, 3, 4 or 5); model 2 multivariable adjusted for covariates in model 1 plus demographic factors including: ethnicity (White, Black, Asian, mixed or other), BMI (<18.5, 18.5–24.99, 25–29.99, ≥30 kg m^−^^2^), education (low (GSEs/O levels/GCSEs or equivalent), medium (NVQ/HND/HNC/A levels/AS levels or equivalent), high (other professional qualifications, college/university degree)) and socioeconomic status (Townsend deprivation index in quintiles); model 3 multivariable adjusted for covariates in model 2 plus lifestyle factors including: smoking status (current, former, never), alcohol intake (<1 g d^−1^, 1–7 g d^−1^, 8–15 g d^−1^, 16+ g d^−1^) and physical activity (MET-h per week in quintiles); model 4 multivariable adjusted for covariates in model 3 plus dietary factors including: intakes of sugary drinks (0 d^−1^, >0–1 d^−1^, >1–2 d^−1^, 2+ d^−1^), cups of coffee (0 d^−1^, >0–1 d^−1^, >1–2 d^−1^, 2+ d^−1^), and red and processed meat, whole grains, refined grains, saturated fatty acids and sodium (all g d^−1^) and energy (kcal d^−1^) (all as quintiles); model 5 multivariable adjusted for covariates in model 4 plus medical history including history of diabetes type 1 or 2 (yes versus no), hypertension (yes versus no) and hypercholesterolaemia (yes versus no), and for analysis of all-cause mortality, further adjustments for prevalent CVD, cancer, respiratory disease and neurodegenerative disease at baseline. For variables where participants could select ‘do not know’ or ‘prefer not to answer’, or for those with missing data, responses were combined into an ‘unknown’ indicator group. The proportional-hazards assumption was confirmed using Schoenfeld residual plots. Absence of multicollinearity among predictors was verified using variance inflation factors. To address concerns that occult chronic diseases in the years preceding diagnosis may have influenced dietary patterns, we conducted sensitivity analysis excluding participants who developed events within 2 years of follow-up. We conducted further sensitivity adjustments for the healthful plant-based diet index in place of other dietary factors in model 5. To assess the influence of flavonoid intakes irrespective of dietary energy, model 5 was rerun without calorie adjustment. To assess the potential independent benefits of quantity and diversity of flavonoid intake on the risk of our outcomes, we report the terms for quantity of flavonoid intake following adjustment for diversity of flavonoid consumption. To evaluate whether the joint effect of quantity and diversity of flavonoid intake was together larger (or smaller) than the combination of the individual parts^42^, likelihood ratio tests were used to compare models with and without interaction terms. We observed and interpreted the magnitude and direction of observed associations through estimated HRs and associated 95% CIs with a HR of 1 indicating no association. All analyses were undertaken using Stata/IC 14.2 (StataCorp) and R statistics (v.4.2.1).

### Reporting summary

Further information on research design is available in the Nature Portfolio Reporting Summary linked to this article.

## Supplementary information

Supplementary Information Supplementary Fig. 1, Tables 1–8, Methods and references.

Reporting Summary

## Data Availability

The UK Biobank dataset used in this study is not publicly available but may be available upon application by bona fide researchers (https://www.ukbiobank.ac.uk/). The UK Nutrient Databank food composition tables are openly accessible (https://www.gov.uk/government/publications/composition-of-foods-integrated-dataset-cofid). The US Department of Agriculture databases for flavonoid (https://agdatacommons.nal.usda.gov/articles/dataset/USDA_Database_for_the_Flavonoid_Content_of_Selected_Foods_Release_3_1_May_2014_/24659802) and proanthocyanin (https://agdatacommons.nal.usda.gov/articles/dataset/USDA_Database_for_the_Proanthocyanidin_Content_of_Selected_Foods_-_2004/25060832) contents in foods are openly accessible.
