# Supplementary material for: High diversity of dietary flavonoid intake is associated with a lower risk of all-cause mortality and major chronic diseases
Source: Nat Food. 2025 Jun 2;6(7):668–80. doi: 10.1038/s43016-025-01176-1 (PMC12283405; doi:10.1038/s43016-025-01176-1)
Supplement: Supplementary file 1 — Supplementary Fig. 1, Tables 1–8, Methods and references. [file 43016_2025_1176_MOESM1_ESM.pdf]

nature food

Supplementary information https://doi.org/10.1038/s43016-025-01176-1

# High diversity of dietary flavonoid intake is associated with a lower risk of all-cause mortality and major chronic diseases

In the format provided by the authors and unedited

**Contents**

Supplementary Figures ........ 2

Supplementary Figure 1. ........ 2

Supplementary Tables ........ 3

Supplementary Table 1 ........ 3

Supplementary Table 2 ........ 3

Supplementary Table 3 ........ 4

Supplementary Table 4 ........ 6

Supplementary Table 5 (Part 1). ........ 7

Supplementary Table 5 (Part 2). ........ 8

Supplementary Table 6 ........ 9

Supplementary Table 7 ........ 10

Supplementary Table 8 ........ 11

Supplementary Methods ........ 12

Supplementary References ........ 13

# Supplementary Figures

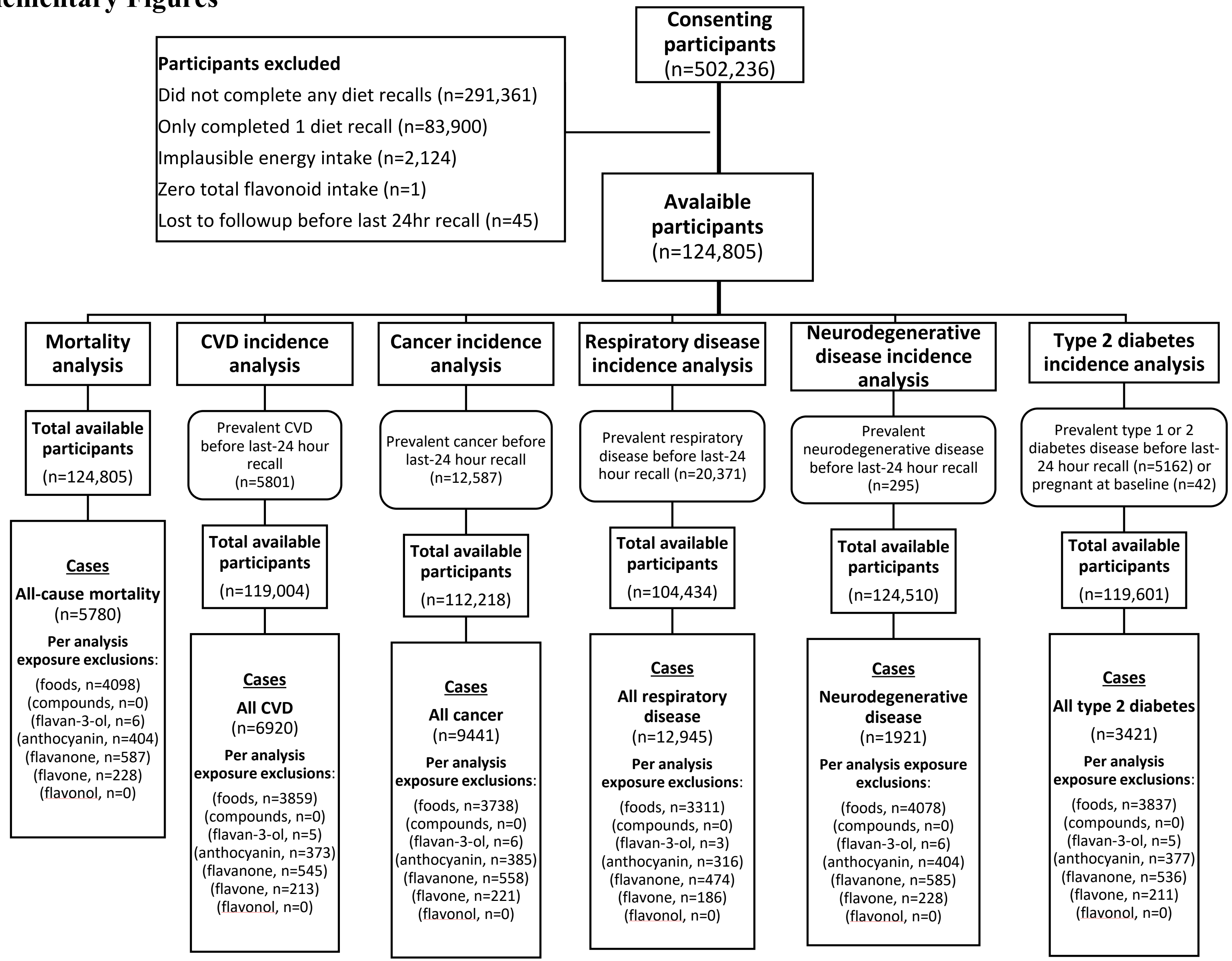


**Supplementary Figure 1.** Flow diagram of UK Biobank participants through the study. CVD, cardiovascular disease.

## Supplementary Tables

**Supplementary Table 1**. Sources of flavonoid intake in the UK Biobank (n=124,805).

| | **Top-most contributing food sources to flavonoid intake** |
|---|---|
| Total flavonoids | Tea (67.3%), Apple (5.8%), Red wine (4.7%), Grapes (1.9%), Berries (1.9%), Dark chocolate (1.2%), Orange & satsuma (1.1%), Orange juice (1.1%), Beans & lentils (0.9%), Pure fruit/vegetable juice (0.9%), Pears (0.9%) |
| Flavan-3-ols | Tea (75.2%), Apple (4.85%), Red wine (3.35%), Pure fruit/vegetable juice (1.2%), Grapes (1.15%), Dark chocolate (1.05%), Herbal tea (1%), Berries (0.9%), Other tea infusions (0.85%), Banana (0.85%) |
| Anthocyanins | Red wine (40.3%), Berries (24.3%), Grapes (14.9%), Apple (2.4%), Fruit yoghurt (2.4%), Other vegetables (2.3%), Smoothies (1.6%), Pears (1.3%), Mixed fruit (1.2%), Cherries (1%) |
| Flavonols | Tea (54.1%), Onion (7.3%), Apple (5.4%), Beer & cider (3.1%), Cabbage & kale (3.1%), Red wine (2.8%), Mixed vegetables (2.4%), Pure fruit/vegetable juice (1.8%), Berries (1.1%), Spinach (1.1%) |
| Flavanones | Orange juice (38.4%), Orange & satsuma (37.7%), Red wine (6%), Grapefruit juice (5.2%), Grapefruit (3.7%), Smoothies (2.2%), White wine (1.5%), Mixed fruit (1.2%), Tomato (0.9%), Jams (0.8%) |
| Flavones | Bell peppers (17.4%), Grapes (10.5%), Red wine (9.3%), Tea (6.8%), Celery (6.4%), Apple (4.8%), Olives (3.2%), Butternut squash (3.2%), Orange & satsuma (2.6%), Courgette (2.5%) |

[1]Food sources do not tally to 100% because minor contributors are not listed. Tea includes both black and green.

**Supplementary Table 2.** Correlations between quantity and diversity flavonoid intake in the UK Biobank

| **Quantity and diversity of** | **Correlation[1]** |
|---|---|
| Total flavonoids | -0.43* |
| Flavonoid-rich foods | 0.23* |
| Flavan-3-ols | -0.26* |
| Anthocyanins | 0.26* |
| Flavonols | 0.46* |
| Flavanones | 0.02* |
| Flavones | 0.14* |

[1]Pearsons correlation. **P* = 0<0.05. Negative correlation indicates decreasing diversity with increasing quantity. Positive correlation indicates increasing diversity with increasing quantity.

**Supplementary Table 3.** Abundance of flavonoid compounds among those with the lowest and highest diversity in the UK Biobank.

| Subclass | Divisions | Compound | Overall average abundance (%)[1] | | Within subclass average abundance (%)[1] | |
|---|---|---|---|---|---|---|
| | | | Low diversity (Q1) | High diversity (Q5) | Low diversity (Q1) | High diversity (Q5) |
| Flavan-3-ol | Monomers | (-)-epicatechin | 2.28 | 4.73 | 2.32 | 5.34 |
| Flavan-3-ol | Monomers | (-)-epicatechin 3-gallate | 4.29 | 3.05 | 4.59 | 4.21 |
| Flavan-3-ol | Monomers | (-)-epigallocatechin | 5.92 | 3.95 | 6.31 | 5.64 |
| Flavan-3-ol | Monomers | (-)-epigallocatechin-3-gallate | 7.20 | 7.14 | 7.63 | 10.10 |
| Flavan-3-ol | Monomers | (+)-catechin | 1.81 | 4.19 | 2.01 | 4.10 |
| Flavan-3-ol | Monomers | (+)-gallocatechin | 0.90 | 0.42 | 0.97 | 0.60 |
| Flavan-3-ol | Proanthocyanidin | Proanthocyanidin dimers | 3.14 | 6.82 | 3.23 | 7.10 |
| Flavan-3-ol | Proanthocyanidin | Proanthocyanidin trimers | 0.85 | 2.72 | 0.82 | 2.97 |
| Flavan-3-ol | Proanthocyanidin | Proanthocyanidin 4-6-mers | 2.04 | 7.40 | 1.93 | 8.37 |
| Flavan-3-ol | Proanthocyanidin | Proanthocyanidin 7-10-mers | 1.22 | 5.30 | 1.12 | 5.86 |
| Flavan-3-ol | Proanthocyanidin | Proanthocyanidin 10-mers | 3.15 | 13.84 | 3.39 | 14.59 |
| Flavan-3-ol | Other derived compounds | Theaflavin | 1.11 | 0.39 | 1.19 | 0.56 |
| Flavan-3-ol | Other derived compounds | theaflavin-3-gallate | 0.87 | 0.30 | 0.94 | 0.44 |
| Flavan-3-ol | Other derived compounds | theaflavin-3,3’-digallate | 1.22 | 0.42 | 1.32 | 0.62 |
| Flavan-3-ol | Other derived compounds | theaflavin-3’-gallate | 1.06 | 0.37 | 1.13 | 0.54 |
| Flavan-3-ol | Other derived compounds | Thearubigins | 56.90 | 19.77 | 61.10 | 28.95 |
| Anthocyanin | n.a. | Cyanidin | 0.26 | 1.57 | 44.65 | 33.94 |
| Anthocyanin | n.a. | Delphinidin | 0.12 | 1.05 | 7.72 | 14.66 |
| Anthocyanin | n.a. | Malvidin | 0.35 | 4.26 | 21.39 | 28.26 |
| Anthocyanin | n.a. | Pelargonidin | 0.11 | 0.48 | 21.25 | 9.94 |
| Anthocyanin | n.a. | Peonidin | 0.05 | 0.50 | 3.42 | 5.57 |
| Anthocyanin | n.a. | Petunidin | 0.05 | 0.68 | 1.58 | 7.62 |
| Flavonol | n.a. | Quercetin | 2.17 | 3.04 | 75.44 | 55.96 |
| Flavonol | n.a. | Myricetin | 0.35 | 0.36 | 5.41 | 8.85 |
| Flavonol | n.a. | Isorhamnetin | 0.07 | 0.23 | 4.54 | 5.31 |
| Flavonol | n.a. | Kaempferol | 1.18 | 0.95 | 14.61 | 29.88 |

| | | | | | | |
|---|---|---|---|---|---|---|
| Flavanones | n.a. | Naringenin | 0.45 | 2.31 | 40.53 | 47.62 |
| Flavanones | n.a. | Eriodictyol | 0.01 | 0.03 | 0.89 | 0.77 |
| Flavanones | n.a. | Hesperetin | 0.81 | 3.46 | 58.59 | 51.61 |
| Flavone | n.a. | Apigenin | 0.01 | 0.07 | 2.5 | 47.1 |
| Flavone | n.a. | Luteolin | 0.05 | 0.19 | 97.5 | 52.9 |

[1]Low and high diversity are matched for quantity of intake. The corresponding visualisation for the overall abundance distribution is located in Figure 1 (Panel D) in the main manuscript.

**Supplementary Table 4.** Abundance of flavonoid-rich foods among those with the lowest and highest diversity in the UK Biobank.

| | Average abundance (%)[1] | |
|---|---|---|
| | **Low Diversity (Q1)** | **High diversity (Q5)** |
| Tea | 85.3 | 37.6 |
| Red wine | 3.6 | 10.7 |
| Apples | 3.5 | 12.7 |
| Berries | 1.3 | 7.8 |
| Grapes | 1.3 | 7.1 |
| Oranges | 2.1 | 12.4 |
| Grapefruit | 0.5 | 0.7 |
| Sweet peppers | 0.5 | 4.2 |
| Onions | 1.5 | 5.7 |
| Dark chocolate | 0.3 | 1.1 |

[1]Low and high diversity are matched for quantity of intake

**Supplementary Table 5 (Part 1).** Diversity of flavonoid-rich food intake and flavonoid subclass intake and risk of all-cause mortality and incidence of major chronic disease in the UK Biobank.[1]

| | Diversity of flavonoid-rich food intake | | | | | Diversity of flavan-3-ol intake | | | | | Diversity of anthocyanin intake | | | | |
|---|---|---|---|---|---|---|---|---|---|---|---|---|---|---|---|
| | Q1 | Q2 | Q3 | Q4 | Q5 | Q1 | Q2 | Q3 | Q4 | Q5 | Q1 | Q2 | Q3 | Q4 | Q5 |
| All-cause mortality | | | | | | | | | | | | | | | |
| Population§ | 24632 | 24578 | 24607 | 24635 | 24573 | 24960 (100%) | 24960 (100%) | 24959 (100%) | 24960 (100%) | 24960 (100%) | 24881 (100%) | 24880 (100%) | 24880 (100%) | 24880 (100%) | 24880 (100%) |
| No. of events | 1328 (5.4%) | 1164 (4.7%) | 1134 (4.6%) | 1067 (4.3%) | 971 (4.0%) | 1286 (5.2%) | 1215 (4.9%) | 1160 (4.6%) | 1066 (4.3%) | 1053 (4.2%) | 1262 (5.1%) | 1197 (4.8%) | 1118 (4.5%) | 1111 (4.5%) | 1076 (4.3%) |
| Hill number* | 1.3 (1, 1.8) | 2 (1.8, 2.4) | 2.7 (2.4, 3) | 3.4 (3, 3.8) | 4.5 (3.8, 9) | 5.0 (1.0, 5.4) | 5.8 (5.4, 6.1) | 6.4 (6.1, 6.8) | 7.3 (6.8, 7.9) | 8.6 (7.9, 10.9) | 2.2 (1.0, 2.7) | 3.0 (2.7, 3.3) | 3.6 (3.3, 3.9) | 4.2 (3.9, 4.5) | 4.7 (4.5, 5.6) |
| HR (95% CI) | | | | | | | | | | | | | | | |
| Model 1 (minimally adjusted) | ref. | **0.86 (0.80, 0.93)** | **0.82 (0.76, 0.89)** | **0.77 (0.71, 0.84)** | **0.71 (0.65, 0.78)** | ref. | **0.89 (0.82, 0.96)** | **0.82 (0.75, 0.88)** | **0.77 (0.71, 0.84)** | **0.76 (0.70, 0.83)** | ref. | 0.93 (0.85, 1.01) | **0.85 (0.78, 0.93)** | **0.88 (0.81, 0.97)** | 0.93 (0.85, 1.02) |
| Model 2 (+ demographics) | ref. | **0.88 (0.82, 0.96)** | **0.85 (0.79, 0.92)** | **0.81 (0.75, 0.88)** | **0.76 (0.69, 0.82)** | ref. | 0.92 (0.85, 1.00) | **0.86 (0.79, 0.93)** | **0.83 (0.76, 0.90)** | **0.82 (0.75, 0.90)** | ref. | 0.95 (0.87, 1.03) | **0.89 (0.81, 0.97)** | 0.92 (0.84, 1.01) | 0.97 (0.88, 1.06) |
| Model 3 (+ lifestyle) | ref. | 0.92 (0.85, 1.00) | **0.90 (0.83, 0.98)** | **0.87 (0.80, 0.95)** | **0.83 (0.76, 0.90)** | ref. | 0.96 (0.89, 1.04) | **0.91 (0.84, 0.99)** | **0.88 (0.81, 0.96)** | **0.89 (0.81, 0.97)** | ref. | 0.97 (0.89, 1.05) | 0.91 (0.84, 1.00) | 0.96 (0.88, 1.05) | 0.99 (0.90, 1.08) |
| Model 4 (+ diet) | ref. | 0.92 (0.85, 1.00) | **0.91 (0.84, 0.99)** | **0.88 (0.81, 0.96)** | **0.84 (0.77, 0.92)** | ref. | 0.97 (0.89, 1.05) | 0.92 (0.85, 1.00) | **0.90 (0.83, 0.98)** | 0.91 (0.83, 1.00) | ref. | 0.97 (0.89, 1.06) | 0.92 (0.85, 1.01) | 0.96 (0.88, 1.05) | 0.99 (0.90, 1.09) |
| Model 5 (+ medical history) | ref. | 0.92 (0.85, 1.00) | **0.91 (0.84, 0.99)** | **0.88 (0.81, 0.96)** | **0.84 (0.76, 0.91)** | ref. | 0.97 (0.89, 1.05) | 0.93 (0.85, 1.01) | **0.91 (0.83, 0.99)** | **0.90 (0.82, 0.99)** | ref. | 0.99 (0.91, 1.08) | 0.94 (0.86, 1.02) | 0.98 (0.89, 1.07) | 1.02 (0.93, 1.11) |
| Sensitivity analysis 1 | ref. | 0.92 (0.85, 1.00) | **0.91 (0.84, 0.99)** | **0.88 (0.81, 0.96)** | **0.83 (0.76, 0.91)** | ref. | 0.97 (0.89, 1.05) | 0.93 (0.85, 1.01) | **0.90 (0.83, 0.98)** | **0.90 (0.82, 0.98)** | ref. | 0.99 (0.91, 1.08) | 0.94 (0.86, 1.02) | 0.98 (0.89, 1.07) | 1.02 (0.93, 1.11) |
| Sensitivity analysis 2 | ref. | 0.93 (0.86, 1.01) | 0.92 (0.85, 1.00) | **0.90 (0.82, 0.98)** | **0.85 (0.78, 0.93)** | ref. | 0.98 (0.90, 1.06) | 0.93 (0.86, 1.02) | 0.92 (0.84, 1.00) | 0.91 (0.83, 1.00) | ref. | 0.98 (0.90, 1.07) | 0.93 (0.86, 1.02) | 0.98 (0.90, 1.07) | 1.01 (0.92, 1.11) |
| Sensitivity analysis 3 | ref. | 0.92 (0.85, 1.00) | **0.90 (0.82, 0.98)** | **0.87 (0.79, 0.95)** | **0.83 (0.76, 0.92)** | ref. | 0.96 (0.88, 1.04) | 0.93 (0.85, 1.01) | **0.90 (0.82, 0.99)** | **0.88 (0.80, 0.97)** | ref. | 0.95 (0.87, 1.04) | **0.88 (0.80, 0.97)** | 0.92 (0.84, 1.02) | 0.98 (0.89, 1.08) |
| Cardiovascular disease incidence | | | | | | | | | | | | | | | |
| Population§ | 24115 | 22899 | 23404 | 23459 | 23470 | 23800 | 23800 | 23799 | 23800 | 23800 | 23727 | 23726 | 23726 | 23726 | 23726 |
| No. of events | 1546 (6.4%) | 1334 (5.8%) | 1315 (5.6%) | 1360 (5.8%) | 1231 (5.2%) | 1475 (6.2%) | 1422 (6.0%) | 1437 (6.0%) | 1334 (5.6%) | 1252 (5.3%) | 1479 (6.2%) | 1464 (6.2%) | 1378 (5.8%) | 1374 (5.8%) | 1193 (5.0%) |
| Hill number* | 1.4 (1, 1.8) | 2 (1.8, 2.4) | 2.7 (2.4, 3) | 3.4 (3, 3.8) | 4.5 (3.8, 9) | 5.0 (1.0, 5.4) | 5.8 (5.4, 6.1) | 6.4 (6.1, 6.8) | 7.3 (6.8, 7.9) | 8.6 (7.9, 10.9) | 2.2 (1.0, 2.7) | 3.0 (2.7, 3.3) | 3.6 (3.3, 3.9) | 4.2 (3.9, 4.5) | 4.7 (4.5, 5.6) |
| HR (95% CI) | | | | | | | | | | | | | | | |
| Model 1 (minimally adjusted) | ref. | **0.91 (0.84, 0.98)** | **0.86 (0.80, 0.93)** | **0.89 (0.83, 0.96)** | **0.83 (0.77, 0.90)** | ref. | **0.92 (0.86, 0.99)** | **0.89 (0.83, 0.96)** | **0.84 (0.78, 0.91)** | **0.81 (0.74, 0.88)** | ref. | 0.98 (0.91, 1.06) | 0.92 (0.85, 1.00) | 0.98 (0.90, 1.06) | 0.94 (0.86, 1.02) |
| Model 2 (+ demographics) | ref. | 0.93 (0.86, 1.00) | **0.89 (0.83, 0.96)** | 0.94 (0.87, 1.01) | **0.88 (0.81, 0.95)** | ref. | 0.95 (0.89, 1.03) | 0.94 (0.87, 1.01) | **0.89 (0.83, 0.96)** | **0.86 (0.80, 0.94)** | ref. | 1.00 (0.93, 1.08) | 0.95 (0.88, 1.03) | 1.01 (0.93, 1.10) | 0.96 (0.88, 1.05) |
| Model 3 (+ lifestyle) | ref. | 0.95 (0.88, 1.02) | **0.92 (0.85, 0.99)** | 0.97 (0.90, 1.05) | 0.92 (0.85, 1.00) | ref. | 0.98 (0.91, 1.05) | 0.97 (0.90, 1.04) | 0.93 (0.86, 1.00) | **0.90 (0.83, 0.98)** | ref. | 1.02 (0.94, 1.10) | 0.97 (0.90, 1.05) | 1.04 (0.95, 1.12) | 0.98 (0.90, 1.06) |
| Model 4 (+ diet) | ref. | 0.95 (0.89, 1.03) | 0.93 (0.86, 1.00) | 0.98 (0.91, 1.06) | 0.94 (0.87, 1.02) | ref. | 0.99 (0.92, 1.07) | 0.99 (0.91, 1.06) | 0.95 (0.88, 1.03) | 0.94 (0.86, 1.02) | ref. | 1.03 (0.95, 1.11) | 0.99 (0.91, 1.07) | 1.05 (0.97, 1.14) | 0.99 (0.91, 1.08) |
| Model 5 (+ medical history) | ref. | 0.95 (0.89, 1.03) | 0.92 (0.86, 1.00) | 0.98 (0.91, 1.06) | 0.94 (0.86, 1.02) | ref. | 1.00 (0.92, 1.07) | 0.99 (0.92, 1.07) | 0.96 (0.89, 1.04) | 0.94 (0.86, 1.02) | ref. | 1.04 (0.96, 1.12) | 1.00 (0.92, 1.08) | 1.06 (0.98, 1.15) | 1.00 (0.92, 1.10) |
| Sensitivity analysis 1 | ref. | 0.95 (0.88, 1.03) | 0.92 (0.86, 1.00) | 0.98 (0.90, 1.05) | 0.93 (0.86, 1.01) | ref. | 0.99 (0.92, 1.07) | 0.99 (0.91, 1.06) | 0.96 (0.88, 1.03) | 0.94 (0.86, 1.02) | ref. | 1.04 (0.96, 1.12) | 1.00 (0.92, 1.08) | 1.06 (0.98, 1.15) | 1.00 (0.92, 1.09) |
| Sensitivity analysis 2 | ref. | 0.96 (0.89, 1.03) | 0.93 (0.86, 1.01) | 0.99 (0.92, 1.07) | 0.96 (0.88, 1.04) | ref. | 1.00 (0.93, 1.08) | 0.99 (0.92, 1.07) | 0.96 (0.89, 1.04) | 0.94 (0.86, 1.02) | ref. | 1.02 (0.95, 1.10) | 0.98 (0.91, 1.07) | 1.05 (0.97, 1.14) | 0.99 (0.91, 1.08) |
| Sensitivity analysis 3 | ref. | 0.95 (0.88, 1.03) | **0.92 (0.84, 0.99)** | 0.97 (0.90, 1.06) | 0.94 (0.86, 1.03) | ref. | 0.98 (0.90, 1.06) | 0.98 (0.90, 1.06) | 0.95 (0.87, 1.03) | 0.94 (0.86, 1.03) | ref. | 0.99 (0.91, 1.08) | 0.97 (0.89, 1.06) | 1.03 (0.95, 1.13) | 0.97 (0.88, 1.06) |
| Type 2 diabetes incidence | | | | | | | | | | | | | | | |
| Population§ | 24293 | 22909 | 23561 | 23578 | 23586 | 23920 | 23919 | 23919 | 23919 | 23919 | 23845 | 23845 | 23844 | 23845 | 23845 |
| No. of events | 849 (3.5%) | 718 (3.1%) | 676 (2.9%) | 587 (2.5%) | 508 (2.2%) | 828 (3.5%) | 737 (3.1%) | 686 (2.9%) | 620 (2.6%) | 550 (2.3%) | 881 (3.7%) | 741 (3.1%) | 612 (2.6%) | 582 (2.4%) | 574 (2.4%) |
| Hill number* | 1.4 (1, 1.8) | 2 (1.8, 2.4) | 2.7 (2.4, 3) | 3.4 (3, 3.8) | 4.5 (3.8, 9) | 5.0 (1.0, 5.4) | 5.8 (5.4, 6.1) | 6.4 (6.1, 6.8) | 7.3 (6.8, 7.9) | 8.6 (7.9, 10.8) | 2.3 (1.0, 2.7) | 3.0 (2.7, 3.3) | 3.6 (3.3, 3.9) | 4.2 (3.9, 4.5) | 4.7 (4.5, 5.6) |
| HR (95% CI) | | | | | | | | | | | | | | | |
| Model 1 (minimally adjusted) | ref. | 0.94 (0.85, 1.04) | **0.86 (0.78, 0.95)** | **0.77 (0.69, 0.85)** | **0.69 (0.62, 0.77)** | ref. | **0.83 (0.75, 0.92)** | **0.73 (0.66, 0.81)** | **0.69 (0.62, 0.76)** | **0.58 (0.52, 0.65)** | ref. | 0.92 (0.83, 1.02) | **0.76 (0.68, 0.85)** | **0.78 (0.69, 0.87)** | **0.84 (0.74, 0.94)** |
| Model 2 (+ demographics) | ref. | 1.01 (0.91, 1.11) | 0.95 (0.86, 1.06) | **0.89 (0.80, 0.99)** | **0.81 (0.72, 0.91)** | ref. | 0.92 (0.83, 1.02) | **0.85 (0.76, 0.94)** | **0.84 (0.75, 0.93)** | **0.73 (0.65, 0.82)** | ref. | 0.99 (0.89, 1.10) | **0.87 (0.78, 0.97)** | 0.89 (0.80, 1.00) | 0.93 (0.83, 1.05) |
| Model 3 (+ lifestyle) | ref. | 1.06 (0.96, 1.17) | 1.03 (0.93, 1.14) | 0.97 (0.87, 1.09) | 0.91 (0.81, 1.02) | ref. | 0.97 (0.88, 1.07) | 0.93 (0.84, 1.03) | 0.91 (0.82, 1.01) | **0.81 (0.72, 0.91)** | ref. | 1.01 (0.91, 1.12) | **0.89 (0.80, 0.99)** | 0.90 (0.80, 1.02) | 0.92 (0.82, 1.04) |
| Model 4 (+ diet) | ref. | 1.07 (0.97, 1.19) | 1.05 (0.95, 1.17) | 1.00 (0.89, 1.12) | 0.94 (0.83, 1.06) | ref. | 0.99 (0.89, 1.10) | 0.97 (0.87, 1.07) | 0.95 (0.85, 1.06) | **0.86 (0.76, 0.97)** | ref. | 1.02 (0.92, 1.13) | 0.89 (0.80, 1.00) | 0.91 (0.81, 1.02) | 0.91 (0.81, 1.03) |
| Model 5 (+ medical history) | ref. | 1.08 (0.97, 1.19) | 1.06 (0.95, 1.17) | 1.00 (0.90, 1.12) | 0.94 (0.83, 1.06) | ref. | 0.99 (0.90, 1.10) | 0.98 (0.88, 1.09) | 0.95 (0.85, 1.06) | **0.87 (0.77, 0.98)** | ref. | 1.01 (0.91, 1.12) | 0.90 (0.80, 1.00) | 0.91 (0.81, 1.02) | 0.92 (0.82, 1.04) |
| Sensitivity analysis 1 | ref. | 1.08 (0.97, 1.19) | 1.06 (0.95, 1.17) | 1.00 (0.90, 1.12) | 0.94 (0.83, 1.05) | ref. | 0.99 (0.89, 1.10) | 0.97 (0.87, 1.08) | 0.95 (0.85, 1.06) | **0.86 (0.77, 0.97)** | ref. | 1.01 (0.91, 1.12) | 0.90 (0.80, 1.00) | 0.91 (0.81, 1.02) | 0.92 (0.82, 1.04) |
| Sensitivity analysis 2 | ref. | 1.09 (0.99, 1.21) | 1.07 (0.97, 1.19) | 1.03 (0.92, 1.15) | 0.97 (0.86, 1.09) | ref. | 0.99 (0.89, 1.09) | 0.96 (0.86, 1.07) | 0.95 (0.85, 1.06) | **0.85 (0.76, 0.96)** | ref. | 1.00 (0.90, 1.11) | 0.90 (0.80, 1.00) | 0.91 (0.81, 1.03) | 0.93 (0.82, 1.04) |
| Sensitivity analysis 3 | ref. | 1.06 (0.95, 1.19) | 1.04 (0.93, 1.17) | 0.96 (0.85, 1.08) | 0.92 (0.81, 1.05) | ref. | 0.96 (0.86, 1.07) | 0.98 (0.87, 1.10) | 0.93 (0.83, 1.05) | **0.84 (0.74, 0.95)** | ref. | 0.97 (0.87, 1.09) | **0.88 (0.78, 0.99)** | 0.89 (0.78, 1.00) | 0.93 (0.82, 1.05) |
| Cancer incidence | | | | | | | | | | | | | | | |
| Population§ | 22131 | 22127 | 22104 | 22122 | 22120 | 22443 | 22442 | 22442 | 22442 | 22443 | 22367 | 22366 | 22367 | 22366 | 22367 |
| No. of events | 1905 (8.6%) | 1821 (8.2%) | 1917 (8.7%) | 1874 (8.5%) | 1798 (8.1%) | 1925 (8.6%) | 1912 (8.5%) | 1911 (8.5%) | 1856 (8.3%) | 1837 (8.2%) | 1846 (8.3%) | 1883 (8.4%) | 1927 (8.6%) | 1912 (8.5%) | 1859 (8.3%) |
| Hill number* | 1.3 (1, 1.8) | 2 (1.8, 2.3) | 2.6 (2.3, 2.9) | 3.4 (2.9, 3.8) | 4.5 (3.8, 9) | 4.9 (1.0, 5.4) | 5.8 (5.4, 6.1) | 6.4 (6.1, 6.8) | 7.3 (6.8, 7.9) | 8.6 (7.9, 10.9) | 2.2 (1.0, 2.7) | 3.0 (2.7, 3.3) | 3.6 (3.3, 3.9) | 4.2 (3.9, 4.5) | 4.7 (4.5, 5.6) |
| HR (95% CI) | | | | | | | | | | | | | | | |
| Model 1 (minimally adjusted) | ref. | 0.94 (0.88, 1.00) | 0.97 (0.91, 1.03) | **0.93 (0.87, 0.99)** | **0.89 (0.84, 0.95)** | ref. | 0.96 (0.90, 1.02) | 0.93 (0.88, 1.00) | **0.89 (0.84, 0.96)** | **0.90 (0.84, 0.96)** | ref. | 0.94 (0.88, 1.00) | 0.95 (0.88, 1.02) | 0.96 (0.89, 1.03) | 0.99 (0.92, 1.06) |
| Model 2 (+ demographics) | ref. | 0.94 (0.88, 1.00) | 0.97 (0.91, 1.04) | 0.94 (0.88, 1.01) | **0.90 (0.85, 0.97)** | ref. | 0.97 (0.91, 1.03) | 0.95 (0.89, 1.01) | **0.91 (0.85, 0.97)** | **0.91 (0.85, 0.98)** | ref. | 0.94 (0.88, 1.01) | 0.96 (0.89, 1.03) | 0.97 (0.90, 1.04) | 0.99 (0.92, 1.07) |
| Model 3 (+ lifestyle) | ref. | 0.95 (0.89, 1.01) | 0.99 (0.92, 1.05) | 0.96 (0.90, 1.02) | **0.92 (0.86, 0.99)** | ref. | 0.98 (0.92, 1.04) | 0.96 (0.90, 1.02) | **0.92 (0.87, 0.99)** | 0.93 (0.87, 1.00) | ref. | 0.94 (0.88, 1.01) | 0.97 (0.90, 1.04) | 0.99 (0.92, 1.06) | 1.01 (0.94, 1.09) |
| Model 4 (+ diet) | ref. | 0.95 (0.89, 1.02) | 0.99 (0.93, 1.06) | 0.96 (0.90, 1.03) | 0.93 (0.87, 1.00) | ref. | 0.97 (0.91, 1.04) | 0.95 (0.89, 1.02) | **0.92 (0.86, 0.99)** | **0.92 (0.86, 0.99)** | ref. | 0.94 (0.88, 1.01) | 0.96 (0.90, 1.03) | 0.98 (0.91, 1.05) | 1.01 (0.94, 1.09) |
| Model 5 (+ medical history) | ref. | 0.95 (0.89, 1.02) | 0.99 (0.93, 1.06) | 0.96 (0.90, 1.03) | 0.93 (0.87, 1.00) | ref. | 0.97 (0.91, 1.04) | 0.95 (0.89, 1.02) | **0.92 (0.86, 0.99)** | **0.92 (0.86, 0.99)** | ref. | 0.94 (0.88, 1.01) | 0.97 (0.90, 1.04) | 0.98 (0.92, 1.06) | 1.01 (0.94, 1.09) |
| Sensitivity analysis 1 | ref. | 0.95 (0.89, 1.02) | 0.99 (0.93, 1.06) | 0.96 (0.90, 1.03) | 0.93 (0.87, 1.00) | ref. | 0.98 (0.92, 1.04) | 0.96 (0.90, 1.02) | **0.93 (0.87, 0.99)** | 0.93 (0.87, 1.00) | ref. | 0.94 (0.88, 1.01) | 0.97 (0.90, 1.04) | 0.98 (0.92, 1.06) | 1.01 (0.94, 1.09) |
| Sensitivity analysis 2 | ref. | 0.95 (0.89, 1.02) | 0.99 (0.93, 1.05) | 0.96 (0.90, 1.03) | 0.93 (0.87, 1.00) | ref. | 0.97 (0.91, 1.04) | 0.95 (0.89, 1.02) | **0.92 (0.86, 0.99)** | **0.92 (0.86, 0.99)** | ref. | 0.94 (0.88, 1.01) | 0.96 (0.90, 1.03) | 0.99 (0.92, 1.06) | 1.01 (0.94, 1.09) |
| Sensitivity analysis 3 | ref. | **0.92 (0.86, 0.99)** | 0.96 (0.90, 1.04) | 0.94 (0.87, 1.01) | **0.89 (0.82, 0.96)** | ref. | 0.97 (0.90, 1.04) | 0.93 (0.87, 1.00) | **0.91 (0.84, 0.98)** | **0.89 (0.82, 0.97)** | ref. | 0.93 (0.86, 1.00) | 0.94 (0.87, 1.02) | 0.97 (0.89, 1.05) | 1.00 (0.92, 1.09) |
| Respiratory disease incidence | | | | | | | | | | | | | | | |
| Population§ | 21063 | 20140 | 20644 | 20551 | 20600 | 20887 | 20886 | 20886 | 20886 | 20886 | 20824 | 20823 | 20824 | 20823 | 20824 |
| No. of events | 2868 (13.6%) | 2540 (12.6%) | 2523 (12.2%) | 2461 (12.0%) | 2330 (11.3%) | 2842 (13.6%) | 2537 (12.1%) | 2633 (12.6%) | 2445 (11.7%) | 2487 (11.9%) | 2861 (13.7%) | 2650 (12.7%) | 2512 (12.1%) | 2470 (11.9%) | 2410 (11.6%) |
| Hill number* | 1.4 (1, 1.8) | 2 (1.8, 2.4) | 2.7 (2.4, 3) | 3.4 (3, 3.8) | 4.5 (3.8, 9) | 5.0 (1.0, 5.4) | 5.8 (5.4, 6.1) | 6.4 (6.1, 6.8) | 7.3 (6.8, 7.9) | 8.6 (7.9, 10.9) | 2.2 (1.0, 2.7) | 3.0 (2.7, 3.3) | 3.6 (3.3, 3.9) | 4.2 (3.9, 4.5) | 4.7 (4.5, 5.6) |
| HR (95% CI) | | | | | | | | | | | | | | | |
| Model 1 (minimally adjusted) | ref. | **0.92 (0.87, 0.97)** | **0.87 (0.83, 0.92)** | **0.85 (0.81, 0.90)** | **0.81 (0.76, 0.85)** | ref. | **0.85 (0.81, 0.90)** | **0.87 (0.82, 0.92)** | **0.82 (0.77, 0.87)** | **0.84 (0.79, 0.89)** | ref. | **0.90 (0.85, 0.96)** | **0.85 (0.80, 0.90)** | **0.86 (0.81, 0.91)** | **0.89 (0.83, 0.94)** |
| Model 2 (+ demographics) | ref. | **0.94 (0.89, 0.99)** | **0.90 (0.85, 0.95)** | **0.89 (0.84, 0.94)** | **0.85 (0.80, 0.90)** | ref. | **0.88 (0.84, 0.93)** | **0.91 (0.86, 0.96)** | **0.87 (0.82, 0.92)** | **0.89 (0.84, 0.95)** | ref. | **0.93 (0.88, 0.98)** | **0.88 (0.83, 0.93)** | **0.89 (0.84, 0.95)** | **0.91 (0.86, 0.97)** |
| Model 3 (+ lifestyle) | ref. | 0.96 (0.91, 1.02) | **0.94 (0.89, 0.99)** | **0.94 (0.88, 0.99)** | **0.90 (0.85, 0.95)** | ref. | **0.91 (0.86, 0.96)** | 0.95 (0.90, 1.00) | **0.91 (0.86, 0.96)** | 0.94 (0.89, 1.00) | ref. | 0.94 (0.89, 1.00) | **0.90 (0.85, 0.95)** | **0.92 (0.87, 0.98)** | **0.93 (0.87, 0.99)** |
| Model 4 (+ diet) | ref. | 0.97 (0.92, 1.02) | 0.95 (0.90, 1.01) | 0.95 (0.90, 1.01) | **0.92 (0.87, 0.98)** | ref. | **0.92 (0.87, 0.97)** | 0.97 (0.91, 1.02) | **0.93 (0.88, 0.98)** | 0.97 (0.91, 1.03) | ref. | 0.94 (0.89, 1.00) | **0.90 (0.85, 0.96)** | **0.93 (0.87, 0.98)** | 0.94 (0.88, 1.00) |
| Model 5 (+ medical history) | ref. | 0.97 (0.92, 1.02) | 0.95 (0.90, 1.00) | 0.95 (0.90, 1.01) | **0.92 (0.87, 0.98)** | ref. | **0.92 (0.87, 0.97)** | 0.97 (0.91, 1.02) | **0.93 (0.88, 0.99)** | 0.97 (0.91, 1.03) | ref. | 0.95 (0.90, 1.01) | **0.91 (0.86, 0.97)** | **0.93 (0.88, 0.99)** | 0.95 (0.89, 1.01) |
| Sensitivity analysis 1 | ref. | 0.97 (0.92, 1.02) | 0.95 (0.90, 1.00) | 0.95 (0.90, 1.00) | **0.92 (0.87, 0.97)** | ref. | **0.92 (0.87, 0.97)** | 0.96 (0.91, 1.02) | **0.93 (0.88, 0.98)** | 0.97 (0.91, 1.03) | ref. | 0.95 (0.90, 1.01) | **0.91 (0.86, 0.97)** | **0.93 (0.88, 0.99)** | 0.95 (0.89, 1.01) |
| Sensitivity analysis 2 | ref. | 0.97 (0.92, 1.03) | 0.95 (0.90, 1.01) | 0.96 (0.90, 1.01) | **0.93 (0.87, 0.99)** | ref. | **0.92 (0.87, 0.97)** | 0.96 (0.91, 1.02) | **0.93 (0.88, 0.99)** | 0.97 (0.91, 1.03) | ref. | 0.94 (0.89, 1.00) | **0.91 (0.85, 0.96)** | **0.93 (0.88, 0.99)** | 0.94 (0.88, 1.00) |
| Sensitivity analysis 3 | ref. | 0.97 (0.92, 1.03) | 0.94 (0.89, 1.00) | 0.94 (0.89, 1.00) | **0.91 (0.85, 0.97)** | ref. | **0.91 (0.85, 0.96)** | 0.96 (0.91, 1.02) | **0.91 (0.86, 0.97)** | 0.95 (0.89, 1.02) | ref. | 0.94 (0.88, 1.00) | **0.89 (0.83, 0.95)** | **0.91 (0.86, 0.98)** | 0.94 (0.88, 1.01) |
| Neurodegenerative disease incidence | | | | | | | | | | | | | | | |
| Population§ | 24575 | 24522 | 24548 | 24589 | 24507 | 24901 | 24901 | 24900 | 24901 | 24901 | 24822 | 24821 | 24821 | 24821 | 24821 |
| No. of events | 364 (1.5%) | 361 (1.5%) | 392 (1.6%) | 402 (1.6%) | 368 (1.5%) | 351 (1.4%) | 367 (1.5%) | 410 (1.6%) | 404 (1.6%) | 389 (1.6%) | 365 (1.5%) | 400 (1.6%) | 402 (1.6%) | 404 (1.6%) | 348 (1.4%) |
| Hill number* | 1.3 (1, 1.8) | 2 (1.8, 2.4) | 2.7 (2.4, 3) | 3.4 (3, 3.8) | 4.5 (3.8, 9) | 5.0 (1.0, 5.4) | 5.8 (5.4, 6.1) | 6.4 (6.1, 6.8) | 7.3 (6.8, 7.9) | 8.6 (7.9, 10.9) | 2.2 (1.0, 2.7) | 3.0 (2.7, 3.3) | 3.6 (3.3, 3.9) | 4.2 (3.9, 4.5) | 4.7 (4.5, 5.6) |
| HR (95% CI) | | | | | | | | | | | | | | | |
| Model 1 (minimally adjusted) | ref. | 0.97 (0.84, 1.13) | 1.01 (0.88, 1.17) | 1.02 (0.88, 1.18) | 0.94 (0.81, 1.09) | ref. | 0.96 (0.83, 1.11) | 1.03 (0.89, 1.19) | 1.01 (0.87, 1.17) | 1.00 (0.86, 1.17) | ref. | 0.95 (0.82, 1.10) | 0.90 (0.77, 1.05) | 0.92 (0.79, 1.08) | 0.88 (0.75, 1.04) |
| Model 2 (+ demographics) | ref. | 0.98 (0.85, 1.14) | 1.03 (0.89, 1.19) | 1.04 (0.90, 1.20) | 0.96 (0.83, 1.12) | ref. | 0.98 (0.84, 1.14) | 1.06 (0.91, 1.22) | 1.04 (0.90, 1.21) | 1.03 (0.88, 1.20) | ref. | 0.96 (0.82, 1.11) | 0.92 (0.78, 1.07) | 0.94 (0.80, 1.10) | 0.89 (0.76, 1.05) |
| Model 3 (+ lifestyle) | ref. | 1.01 (0.87, 1.17) | 1.05 (0.91, 1.22) | 1.06 (0.92, 1.23) | 0.99 (0.85, 1.16) | ref. | 1.02 (0.88, 1.18) | 1.09 (0.94, 1.26) | 1.08 (0.93, 1.26) | 1.06 (0.91, 1.24) | ref. | 0.95 (0.82, 1.11) | 0.92 (0.79, 1.08) | 0.95 (0.81, 1.12) | 0.89 (0.76, 1.06) |
| Model 4 (+ diet) | ref. | 1.01 (0.87, 1.17) | 1.05 (0.90, 1.21) | 1.06 (0.91, 1.23) | 0.98 (0.84, 1.15) | ref. | 1.02 (0.88, 1.18) | 1.10 (0.94, 1.28) | 1.09 (0.94, 1.27) | 1.07 (0.91, 1.26) | ref. | 0.97 (0.84, 1.13) | 0.94 (0.81, 1.10) | 0.97 (0.82, 1.14) | 0.91 (0.77, 1.07) |
| Model 5 (+ medical history) | ref. | 1.00 (0.87, 1.17) | 1.04 (0.90, 1.21) | 1.05 (0.91, 1.22) | 0.99 (0.84, 1.15) | ref. | 1.02 (0.88, 1.19) | 1.09 (0.94, 1.27) | 1.10 (0.94, 1.28) | 1.08 (0.92, 1.27) | ref. | 0.98 (0.84, 1.15) | 0.95 (0.81, 1.11) | 0.98 (0.83, 1.15) | 0.92 (0.78, 1.09) |
| Sensitivity analysis 1 | ref. | 1.00 (0.87, 1.17) | 1.04 (0.90, 1.21) | 1.05 (0.91, 1.22) | 0.99 (0.84, 1.15) | ref. | 1.02 (0.88, 1.19) | 1.09 (0.94, 1.27) | 1.10 (0.94, 1.28) | 1.08 (0.92, 1.27) | ref. | 0.98 (0.84, 1.15) | 0.95 (0.81, 1.11) | 0.98 (0.83, 1.15) | 0.93 (0.78, 1.09) |
| Sensitivity analysis 2 | ref. | 1.01 (0.87, 1.17) | 1.05 (0.91, 1.22) | 1.07 (0.92, 1.24) | 1.00 (0.86, 1.18) | ref. | 1.03 (0.89, 1.20) | 1.10 (0.95, 1.28) | 1.11 (0.95, 1.30) | 1.09 (0.93, 1.29) | ref. | 0.97 (0.83, 1.12) | 0.94 (0.81, 1.10) | 0.97 (0.83, 1.14) | 0.91 (0.77, 1.08) |
| Sensitivity analysis 3 | ref. | 0.97 (0.83, 1.13) | 1.04 (0.89, 1.21) | 1.05 (0.90, 1.22) | 0.99 (0.84, 1.16) | ref. | 1.00 (0.86, 1.17) | 1.09 (0.94, 1.27) | 1.11 (0.95, 1.30) | 1.08 (0.92, 1.28) | ref. | 0.97 (0.83, 1.14) | 0.95 (0.81, 1.11) | 0.97 (0.82, 1.14) | 0.92 (0.77, 1.09) |

[1]Hazard ratios (95% CI) for risk of all-cause mortality and major chronic diseases obtained from Cox proportional hazards models with age as the underlying timescale. All models considering diversity of servings of flavonoid-rich food intake were adjusted for quantity of servings of the same flavonoid-rich foods. All models considering flavonoid subclasses were additionally individually adjusted for quantity of intake of the specific flavonoid subclass of interest. Model 1, minimally adjusted for: sex, region of residence and number of dietary recalls. Model 2, multivariable adjusted for model 1 plus demographic factors including: ethnicity, BMI, education, social economic status (Townsend deprivation index). Model 3, multivariable adjusted for model 2 plus lifestyle factors including smoking, physical activity and alcohol intake. Model 4, multivariable adjusted for model 3 plus dietary factors including intakes (g/d) of red and processed meat, refined grains, whole grains, sugary drinks, coffee, saturated fatty acids, sodium and (kcal/d) energy. Model 5, multivariable adjusted for model 4 plus medical history including history of diabetes mellitus, hypertension and hypercholesterolemia and for analysis of all-cause mortality, models were further adjusted for prevalent CVD, cancer, respiratory disease, and neurodegenerative disease at baseline. Sensitivity analysis 1: excludes energy intake from Model 5. Sensitivity analysis 2: adjusts for a healthful plant-based diet index in place of other dietary covariates in Model 5 retaining energy intake. Sensitivity analysis 3: excludes participants who had an event in the first 2 years of follow-up using Model 5; case event numbers listed in the table do not apply to this analysis. §Sample sizes differ between analyses due to differing per analysis exclusions. *Intake shows flavonoid quantity (mg/d) and diversity as Hill's effective numbers. Median; min to max [all such values]. Intake quintiles are mutually exclusive.

**Supplementary Table 5 (Part 2).** Diversity of flavonoid-rich food intake and flavonoid subclass intake and risk of all-cause mortality and incidence of major chronic disease in the UK Biobank.[1]

| | Diversity of flavonol intake | | | | | Diversity of flavanone intake | | | | | Diversity of flavone intake | | | | |
|---|---|---|---|---|---|---|---|---|---|---|---|---|---|---|---|
| | **Q1** | **Q2** | **Q3** | **Q4** | **Q5** | **Q1** | **Q2** | **Q3** | **Q4** | **Q5** | **Q1** | **Q2** | **Q3** | **Q4** | **Q5** |
| All-cause mortality | | | | | | | | | | | | | | | |
| Population§ | 24961 (100%) | 24961 (100%) | 24961 (100%) | 24961 (100%) | 24961 (100%) | 24844 | 24843 | 24844 | 24843 | 24844 | 24916 | 24915 | 24915 | 24915 | 24916 |
| No. of events | 1164 (4.7%) | 1030 (4.1%) | 1150 (4.6%) | 1185 (4.7%) | 1251 (5.0%) | 1246 (5.0%) | 1131 (4.6%) | 1107 (4.5%) | 1134 (4.6%) | 1128 (4.5%) | 1229 (4.9%) | 1125 (4.5%) | 1110 (4.5%) | 1146 (4.6%) | 1159 (4.7%) |
| Hill number* | 2.1 (1.0, 2.4) | 2.5 (2.4, 2.5) | 2.6 (2.5, 2.6) | 2.6 (2.6, 2.7) | 2.8 (2.7, 3.6) | 1.6 (1.0, 1.7) | 1.8 (1.7, 1.9) | 1.9 (1.9, 2.0) | 2.0 (2.0, 2.0) | 2.1 (2.0, 3.0) | 1.1 (1.0, 1.2) | 1.4 (1.2, 1.5) | 1.6 (1.5, 1.7) | 1.8 (1.7, 1.9) | 2.0 (1.9, 2.0) |
| HR (95% CI) | | | | | | | | | | | | | | | |
| Model 1 (minimally adjusted) | ref. | **0.88 (0.80, 0.96)** | 0.99 (0.90, 1.08) | 0.99 (0.91, 1.09) | 1.01 (0.93, 1.11) | ref. | **0.84 (0.78, 0.91)** | **0.80 (0.74, 0.87)** | **0.85 (0.79, 0.93)** | **0.81 (0.74, 0.88)** | ref. | **0.91 (0.84, 0.99)** | **0.89 (0.82, 0.97)** | **0.89 (0.82, 0.97)** | **0.89 (0.82, 0.97)** |
| Model 2 (+ demographics) | ref. | **0.89 (0.81, 0.97)** | 0.99 (0.90, 1.08) | 0.99 (0.90, 1.08) | 1.00 (0.92, 1.10) | ref. | **0.87 (0.80, 0.95)** | **0.82 (0.75, 0.89)** | **0.87 (0.80, 0.95)** | **0.84 (0.78, 0.91)** | ref. | 0.93 (0.86, 1.01) | 0.93 (0.85, 1.01) | 0.93 (0.85, 1.00) | **0.91 (0.84, 0.99)** |
| Model 3 (+ lifestyle) | ref. | **0.90 (0.82, 0.98)** | 0.98 (0.90, 1.08) | 0.98 (0.89, 1.07) | 1.00 (0.91, 1.09) | ref. | **0.91 (0.83, 0.98)** | **0.85 (0.78, 0.92)** | **0.90 (0.83, 0.98)** | **0.88 (0.81, 0.95)** | ref. | 0.95 (0.87, 1.03) | 0.96 (0.88, 1.04) | 0.95 (0.87, 1.03) | 0.93 (0.86, 1.01) |
| Model 4 (+ diet) | ref. | **0.89 (0.82, 0.98)** | 0.97 (0.88, 1.06) | 0.97 (0.88, 1.06) | 0.99 (0.90, 1.08) | ref. | **0.91 (0.84, 0.99)** | **0.85 (0.79, 0.93)** | **0.92 (0.84, 0.99)** | **0.89 (0.82, 0.96)** | ref. | 0.95 (0.88, 1.03) | 0.96 (0.88, 1.04) | 0.95 (0.88, 1.04) | 0.94 (0.86, 1.02) |
| Model 5 (+ medical history) | ref. | **0.89 (0.81, 0.98)** | 0.98 (0.89, 1.07) | 0.96 (0.88, 1.06) | 0.98 (0.89, 1.07) | ref. | **0.90 (0.83, 0.98)** | **0.84 (0.77, 0.91)** | **0.90 (0.83, 0.98)** | **0.88 (0.81, 0.96)** | ref. | 0.95 (0.88, 1.03) | 0.95 (0.88, 1.04) | 0.95 (0.87, 1.03) | 0.93 (0.85, 1.01) |
| Sensitivity analysis 1 | ref. | **0.89 (0.82, 0.98)** | 0.98 (0.89, 1.07) | 0.97 (0.88, 1.06) | 0.98 (0.89, 1.07) | ref. | **0.90 (0.83, 0.98)** | **0.84 (0.77, 0.91)** | **0.90 (0.83, 0.98)** | **0.88 (0.81, 0.96)** | ref. | 0.95 (0.88, 1.03) | 0.95 (0.88, 1.04) | 0.95 (0.87, 1.03) | 0.93 (0.85, 1.01) |
| Sensitivity analysis 2 | ref. | **0.89 (0.81, 0.97)** | 0.97 (0.88, 1.06) | 0.95 (0.87, 1.05) | 0.97 (0.88, 1.06) | ref. | **0.90 (0.83, 0.98)** | **0.85 (0.78, 0.92)** | 0.92 (0.85, 1.00) | **0.90 (0.82, 0.97)** | ref. | 0.95 (0.88, 1.03) | 0.95 (0.87, 1.03) | 0.94 (0.87, 1.02) | 0.92 (0.84, 1.00) |
| Sensitivity analysis 3 | ref. | **0.89 (0.81, 0.98)** | 0.98 (0.89, 1.08) | 0.94 (0.85, 1.04) | 0.96 (0.87, 1.06) | ref. | 0.92 (0.84, 1.00) | **0.85 (0.78, 0.94)** | 0.93 (0.85, 1.01) | **0.87 (0.80, 0.95)** | ref. | 0.96 (0.88, 1.05) | 0.93 (0.85, 1.02) | 0.94 (0.86, 1.03) | **0.91 (0.83, 0.99)** |
| Cardiovascular disease incidence | | | | | | | | | | | | | | | |
| Population§ | 23801 | 23801 | 23800 | 23801 | 23801 | 23692 | 23692 | 23691 | 23692 | 23692 | 23759 | 23758 | 23758 | 23758 | 23758 |
| No. of events | 1274 (5.4%) | 1336 (5.6%) | 1435 (6.0%) | 1418 (6.0%) | 1457 (6.1%) | 1379 (5.8%) | 1302 (5.5%) | 1442 (6.1%) | 1366 (5.8%) | 1385 (5.8%) | 1439 (6.1%) | 1316 (5.5%) | 1392 (5.9%) | 1357 (5.7%) | 1390 (5.9%) |
| Hill number* | 2.1 (1.0, 2.4) | 2.5 (2.4, 2.5) | 2.6 (2.5, 2.6) | 2.6 (2.6, 2.7) | 2.8 (2.7, 3.6) | 1.6 (1.0, 1.7) | 1.8 (1.7, 1.9) | 1.9 (1.9, 2.0) | 2.0 (2.0, 2.0) | 2.1 (2.0, 3.0) | 1.1 (1.0, 1.2) | 1.4 (1.2, 1.5) | 1.6 (1.5, 1.7) | 1.8 (1.7, 1.9) | 2.0 (1.9, 2.0) |
| HR (95% CI) | | | | | | | | | | | | | | | |
| Model 1 (minimally adjusted) | ref. | 1.02 (0.93, 1.10) | 1.10 (1.01, 1.20) | 1.06 (0.98, 1.16) | 1.05 (0.97, 1.15) | ref. | **0.90 (0.84, 0.98)** | 0.98 (0.91, 1.06) | 0.97 (0.90, 1.05) | 0.93 (0.86, 1.00) | ref. | 0.92 (0.86, 1.00) | 0.96 (0.89, 1.04) | **0.90 (0.84, 0.97)** | **0.91 (0.84, 0.98)** |
| Model 2 (+ demographics) | ref. | 1.02 (0.94, 1.11) | 1.09 (1.00, 1.19) | 1.05 (0.97, 1.14) | 1.04 (0.95, 1.13) | ref. | 0.93 (0.86, 1.00) | 0.99 (0.92, 1.07) | 0.99 (0.92, 1.07) | 0.96 (0.89, 1.04) | ref. | 0.94 (0.87, 1.01) | 0.99 (0.92, 1.07) | 0.93 (0.86, 1.00) | 0.93 (0.86, 1.00) |
| Model 3 (+ lifestyle) | ref. | 1.03 (0.95, 1.12) | 1.09 (1.00, 1.19) | 1.05 (0.97, 1.15) | 1.04 (0.95, 1.13) | ref. | 0.95 (0.88, 1.03) | 1.01 (0.94, 1.09) | 1.01 (0.94, 1.09) | 0.99 (0.91, 1.06) | ref. | 0.95 (0.88, 1.02) | 1.01 (0.94, 1.09) | 0.94 (0.87, 1.02) | 0.93 (0.86, 1.01) |
| Model 4 (+ diet) | ref. | 1.04 (0.96, 1.13) | 1.09 (1.00, 1.19) | 1.06 (0.97, 1.15) | 1.04 (0.95, 1.13) | ref. | 0.96 (0.89, 1.04) | 1.02 (0.95, 1.10) | 1.02 (0.95, 1.10) | 1.00 (0.93, 1.08) | ref. | 0.95 (0.88, 1.03) | 1.02 (0.94, 1.10) | 0.95 (0.88, 1.03) | 0.94 (0.87, 1.02) |
| Model 5 (+ medical history) | ref. | 1.04 (0.96, 1.13) | 1.10 (1.01, 1.20) | 1.06 (0.97, 1.16) | 1.04 (0.96, 1.13) | ref. | 0.96 (0.88, 1.03) | 1.01 (0.94, 1.09) | 1.01 (0.94, 1.09) | 1.00 (0.93, 1.08) | ref. | 0.95 (0.88, 1.03) | 1.02 (0.94, 1.10) | 0.95 (0.88, 1.03) | 0.94 (0.87, 1.01) |
| Sensitivity analysis 1 | ref. | 1.04 (0.96, 1.13) | 1.10 (1.01, 1.20) | 1.06 (0.97, 1.16) | 1.04 (0.96, 1.14) | ref. | 0.95 (0.88, 1.03) | 1.01 (0.94, 1.09) | 1.01 (0.94, 1.09) | 1.00 (0.93, 1.08) | ref. | 0.95 (0.88, 1.03) | 1.02 (0.94, 1.10) | 0.95 (0.88, 1.03) | 0.94 (0.87, 1.01) |
| Sensitivity analysis 2 | ref. | 1.03 (0.95, 1.12) | 1.08 (0.99, 1.17) | 1.04 (0.95, 1.13) | 1.02 (0.94, 1.12) | ref. | 0.95 (0.88, 1.03) | 1.02 (0.95, 1.11) | 1.03 (0.95, 1.11) | 1.01 (0.94, 1.09) | ref. | 0.96 (0.89, 1.03) | 1.02 (0.94, 1.09) | 0.94 (0.87, 1.02) | 0.93 (0.86, 1.00) |
| Sensitivity analysis 3 | ref. | 1.05 (0.96, 1.14) | 1.10 (1.00, 1.20) | 1.06 (0.97, 1.16) | 1.06 (0.96, 1.16) | ref. | 0.95 (0.88, 1.04) | 1.00 (0.92, 1.09) | 0.99 (0.92, 1.08) | 0.97 (0.89, 1.05) | ref. | 0.95 (0.88, 1.03) | 1.01 (0.93, 1.10) | 0.95 (0.88, 1.03) | 0.93 (0.86, 1.01) |
| Type 2 diabetes incidence | | | | | | | | | | | | | | | |
| Population§ | 23921 | 23920 | 23920 | 23920 | 23920 | 23813 | 23813 | 23813 | 23813 | 23813 | 23878 | 23878 | 23878 | 23878 | 23878 |
| No. of events | 704 (2.9%) | 650 (2.7%) | 692 (2.9%) | 658 (2.8%) | 717 (3.0%) | 801 (3.4%) | 662 (2.8%) | 682 (2.9%) | 622 (2.6%) | 620 (2.6%) | 844 (3.5%) | 679 (2.8%) | 646 (2.7%) | 617 (2.6%) | 619 (2.6%) |
| Hill number* | 2.1 (1.0, 2.4) | 2.5 (2.4, 2.5) | 2.6 (2.5, 2.6) | 2.6 (2.6, 2.7) | 2.8 (2.7, 3.6) | 1.6 (1.0, 1.7) | 1.8 (1.7, 1.9) | 1.9 (1.9, 2.0) | 2.0 (2.0, 2.0) | 2.1 (2.0, 3.0) | 1.1 (1.0, 1.2) | 1.4 (1.2, 1.5) | 1.6 (1.5, 1.7) | 1.9 (1.7, 1.9) | 2.0 (1.9, 2.0) |
| HR (95% CI) | | | | | | | | | | | | | | | |
| Model 1 (minimally adjusted) | ref. | 1.01 (0.90, 1.13) | 1.12 (0.99, 1.26) | 1.05 (0.94, 1.19) | 1.11 (0.99, 1.25) | ref. | **0.82 (0.74, 0.91)** | **0.83 (0.75, 0.92)** | **0.78 (0.70, 0.87)** | **0.73 (0.66, 0.81)** | ref. | **0.83 (0.75, 0.91)** | **0.81 (0.73, 0.90)** | **0.75 (0.67, 0.83)** | **0.74 (0.67, 0.82)** |
| Model 2 (+ demographics) | ref. | 1.03 (0.92, 1.15) | 1.10 (0.98, 1.23) | 1.02 (0.90, 1.15) | 1.07 (0.95, 1.20) | ref. | 0.92 (0.83, 1.02) | **0.88 (0.79, 0.98)** | **0.84 (0.76, 0.94)** | **0.83 (0.75, 0.93)** | ref. | **0.88 (0.80, 0.98)** | **0.89 (0.81, 0.99)** | **0.82 (0.74, 0.91)** | **0.80 (0.72, 0.89)** |
| Model 3 (+ lifestyle) | ref. | 1.05 (0.94, 1.18) | 1.11 (0.99, 1.25) | 1.05 (0.94, 1.19) | 1.11 (0.99, 1.25) | ref. | 0.99 (0.89, 1.10) | 0.93 (0.84, 1.04) | 0.90 (0.81, 1.00) | **0.89 (0.80, 0.99)** | ref. | 0.91 (0.82, 1.00) | 0.94 (0.85, 1.05) | **0.88 (0.79, 0.97)** | **0.86 (0.77, 0.96)** |
| Model 4 (+ diet) | ref. | 1.05 (0.94, 1.18) | 1.09 (0.97, 1.23) | 1.04 (0.92, 1.18) | 1.10 (0.98, 1.24) | ref. | 1.01 (0.91, 1.12) | 0.94 (0.85, 1.05) | 0.91 (0.82, 1.02) | 0.91 (0.81, 1.01) | ref. | 0.92 (0.83, 1.02) | 0.96 (0.87, 1.07) | **0.89 (0.80, 0.99)** | **0.88 (0.79, 0.98)** |
| Model 5 (+ medical history) | ref. | 1.05 (0.94, 1.18) | 1.09 (0.97, 1.23) | 1.04 (0.92, 1.17) | 1.09 (0.97, 1.23) | ref. | 1.00 (0.90, 1.11) | 0.93 (0.84, 1.03) | 0.90 (0.81, 1.01) | 0.91 (0.82, 1.01) | ref. | 0.91 (0.83, 1.01) | 0.96 (0.86, 1.07) | **0.89 (0.80, 0.99)** | **0.87 (0.78, 0.97)** |
| Sensitivity analysis 1 | ref. | 1.06 (0.94, 1.18) | 1.10 (0.97, 1.23) | 1.04 (0.92, 1.17) | 1.09 (0.97, 1.23) | ref. | 0.99 (0.89, 1.11) | 0.93 (0.83, 1.03) | 0.90 (0.81, 1.00) | 0.91 (0.81, 1.01) | ref. | 0.91 (0.83, 1.01) | 0.96 (0.86, 1.06) | **0.89 (0.80, 0.99)** | **0.87 (0.78, 0.97)** |
| Sensitivity analysis 2 | ref. | 1.04 (0.93, 1.17) | 1.09 (0.97, 1.23) | 1.03 (0.91, 1.16) | 1.08 (0.96, 1.21) | ref. | 1.00 (0.90, 1.11) | 0.96 (0.87, 1.07) | 0.94 (0.85, 1.05) | 0.93 (0.83, 1.04) | ref. | 0.91 (0.83, 1.01) | 0.95 (0.85, 1.05) | **0.87 (0.78, 0.97)** | **0.84 (0.75, 0.93)** |
| Sensitivity analysis 3 | ref. | 1.02 (0.90, 1.15) | 1.08 (0.96, 1.23) | 1.02 (0.89, 1.16) | 1.08 (0.95, 1.23) | ref. | 0.92 (0.82, 1.04) | 0.89 (0.79, 1.00) | **0.85 (0.75, 0.95)** | **0.86 (0.77, 0.97)** | ref. | 0.91 (0.82, 1.02) | 0.96 (0.86, 1.07) | **0.88 (0.79, 0.99)** | **0.85 (0.76, 0.96)** |
| Cancer incidence | | | | | | | | | | | | | | | |
| Population§ | 22444 | 22443 | 22444 | 22443 | 22444 | 22332 | 22332 | 22332 | 22332 | 22332 | 22400 | 22399 | 22399 | 22399 | 22400 |
| No. of events | 1787 (8.0%) | 1847 (8.2%) | 1905 (8.5%) | 1922 (8.6%) | 1980 (8.8%) | 1820 (8.1%) | 1854 (8.3%) | 1964 (8.8%) | 1911 (8.6%) | 1859 (8.3%) | 1837 (8.2%) | 1888 (8.4%) | 1856 (8.3%) | 1918 (8.6%) | 1933 (8.6%) |
| Hill number* | 2.1 (1.0, 2.4) | 2.5 (2.4, 2.5) | 2.6 (2.5, 2.6) | 2.6 (2.6, 2.7) | 2.8 (2.7, 3.6) | 1.6 (1.0, 1.7) | 1.8 (1.7, 1.9) | 1.9 (1.9, 2.0) | 2.0 (2.0, 2.0) | 2.1 (2.0, 3.0) | 1.1 (1.0, 1.2) | 1.4 (1.2, 1.5) | 1.6 (1.5, 1.7) | 1.8 (1.7, 1.9) | 2.0 (1.9, 2.0) |
| HR (95% CI) | | | | | | | | | | | | | | | |
| Model 1 (minimally adjusted) | ref. | 0.97 (0.91, 1.04) | 1.00 (0.93, 1.08) | 0.99 (0.92, 1.07) | 1.00 (0.93, 1.07) | ref. | 0.95 (0.89, 1.01) | 0.98 (0.92, 1.05) | 0.98 (0.91, 1.04) | **0.92 (0.86, 0.98)** | ref. | 1.01 (0.95, 1.08) | 0.98 (0.92, 1.04) | 0.99 (0.93, 1.05) | 0.98 (0.92, 1.04) |
| Model 2 (+ demographics) | ref. | 0.98 (0.91, 1.05) | 1.00 (0.93, 1.08) | 0.99 (0.92, 1.06) | 1.00 (0.93, 1.07) | ref. | 0.95 (0.89, 1.02) | 0.99 (0.92, 1.05) | 0.98 (0.92, 1.05) | **0.93 (0.87, 0.99)** | ref. | 1.01 (0.95, 1.08) | 0.98 (0.92, 1.05) | 0.99 (0.93, 1.06) | 0.98 (0.92, 1.04) |
| Model 3 (+ lifestyle) | ref. | 0.98 (0.91, 1.05) | 1.00 (0.93, 1.07) | 0.98 (0.91, 1.05) | 0.98 (0.91, 1.06) | ref. | 0.95 (0.89, 1.02) | 0.99 (0.93, 1.06) | 0.98 (0.92, 1.05) | 0.94 (0.88, 1.00) | ref. | 1.02 (0.95, 1.08) | 0.98 (0.92, 1.05) | 0.98 (0.92, 1.05) | 0.96 (0.90, 1.03) |
| Model 4 (+ diet) | ref. | 0.97 (0.91, 1.04) | 0.99 (0.92, 1.07) | 0.97 (0.90, 1.04) | 0.98 (0.91, 1.05) | ref. | 0.95 (0.89, 1.02) | 0.99 (0.93, 1.06) | 0.98 (0.92, 1.05) | **0.93 (0.87, 0.99)** | ref. | 1.01 (0.95, 1.08) | 0.98 (0.92, 1.05) | 0.98 (0.92, 1.05) | 0.96 (0.90, 1.03) |
| Model 5 (+ medical history) | ref. | 0.97 (0.91, 1.05) | 0.99 (0.92, 1.07) | 0.97 (0.90, 1.05) | 0.98 (0.91, 1.05) | ref. | 0.95 (0.89, 1.01) | 0.99 (0.92, 1.05) | 0.98 (0.92, 1.05) | **0.93 (0.87, 0.99)** | ref. | 1.01 (0.95, 1.08) | 0.98 (0.92, 1.05) | 0.98 (0.92, 1.05) | 0.96 (0.90, 1.02) |
| Sensitivity analysis 1 | ref. | 0.97 (0.91, 1.04) | 0.99 (0.92, 1.06) | 0.97 (0.90, 1.04) | 0.98 (0.91, 1.05) | ref. | 0.95 (0.89, 1.01) | 0.99 (0.92, 1.05) | 0.98 (0.92, 1.05) | **0.93 (0.87, 0.99)** | ref. | 1.01 (0.95, 1.08) | 0.98 (0.92, 1.05) | 0.98 (0.92, 1.04) | 0.96 (0.90, 1.02) |
| Sensitivity analysis 2 | ref. | 0.98 (0.91, 1.05) | 0.99 (0.93, 1.07) | 0.97 (0.91, 1.05) | 0.98 (0.91, 1.06) | ref. | 0.95 (0.89, 1.02) | 0.99 (0.93, 1.06) | 0.99 (0.92, 1.05) | 0.93 (0.87, 1.00) | ref. | 1.02 (0.95, 1.08) | 0.98 (0.92, 1.05) | 0.98 (0.92, 1.05) | 0.96 (0.90, 1.03) |
| Sensitivity analysis 3 | ref. | 0.96 (0.89, 1.04) | 1.00 (0.92, 1.09) | 0.98 (0.90, 1.06) | 0.96 (0.89, 1.05) | ref. | 0.96 (0.89, 1.03) | 0.98 (0.91, 1.05) | 0.99 (0.92, 1.06) | **0.91 (0.85, 0.99)** | ref. | 1.02 (0.94, 1.09) | 0.98 (0.91, 1.05) | 1.00 (0.93, 1.07) | 0.97 (0.90, 1.04) |
| Respiratory disease incidence | | | | | | | | | | | | | | | |
| Population§ | 20887 | 20887 | 20886 | 20887 | 20887 | 20792 | 20792 | 20792 | 20792 | 20792 | 20850 | 20849 | 20850 | 20849 | 20850 |
| No. of events | 2577 (12.3%) | 2466 (11.8%) | 2569 (12.3%) | 2614 (12.5%) | 2719 (13.0%) | 2649 (12.7%) | 2474 (11.9%) | 2640 (12.7%) | 2590 (12.5%) | 2521 (12.1%) | 2732 (13.1%) | 2507 (12.0%) | 2476 (11.9%) | 2554 (12.3%) | 2654 (12.7%) |
| Hill number* | 2.2 (1.0, 2.4) | 2.5 (2.4, 2.5) | 2.6 (2.5, 2.6) | 2.6 (2.6, 2.7) | 2.8 (2.7, 3.6) | 1.6 (1.0, 1.7) | 1.8 (1.7, 1.9) | 1.9 (1.9, 2.0) | 2.0 (2.0, 2.0) | 2.1 (2.0, 3.0) | 1.1 (1.0, 1.2) | 1.4 (1.2, 1.5) | 1.6 (1.5, 1.7) | 1.9 (1.7, 1.9) | 2.0 (1.9, 2.0) |
| HR (95% CI) | | | | | | | | | | | | | | | |
| Model 1 (minimally adjusted) | ref. | 0.94 (0.89, 1.00) | 0.99 (0.93, 1.05) | 1.00 (0.94, 1.06) | 1.00 (0.94, 1.06) | ref. | **0.89 (0.84, 0.94)** | **0.93 (0.88, 0.98)** | **0.93 (0.88, 0.98)** | **0.87 (0.83, 0.92)** | ref. | **0.91 (0.86, 0.96)** | **0.89 (0.85, 0.94)** | **0.91 (0.86, 0.96)** | **0.94 (0.89, 0.99)** |
| Model 2 (+ demographics) | ref. | 0.95 (0.89, 1.00) | 0.99 (0.93, 1.05) | 0.99 (0.93, 1.05) | 0.99 (0.93, 1.05) | ref. | **0.92 (0.87, 0.97)** | 0.94 (0.89, 1.00) | 0.95 (0.90, 1.00) | **0.90 (0.85, 0.95)** | ref. | **0.93 (0.88, 0.98)** | **0.92 (0.87, 0.98)** | **0.93 (0.88, 0.99)** | 0.95 (0.90, 1.01) |
| Model 3 (+ lifestyle) | ref. | 0.96 (0.90, 1.02) | 0.98 (0.93, 1.05) | 0.98 (0.92, 1.05) | 0.98 (0.92, 1.04) | ref. | **0.94 (0.89, 0.99)** | 0.96 (0.91, 1.02) | 0.97 (0.92, 1.02) | **0.93 (0.88, 0.98)** | ref. | **0.94 (0.89, 0.99)** | 0.94 (0.89, 1.00) | 0.95 (0.90, 1.00) | 0.96 (0.91, 1.02) |
| Model 4 (+ diet) | ref. | 0.96 (0.90, 1.02) | 0.97 (0.92, 1.04) | 0.98 (0.92, 1.04) | 0.98 (0.92, 1.04) | ref. | 0.94 (0.89, 1.00) | 0.97 (0.92, 1.03) | 0.98 (0.92, 1.03) | **0.94 (0.89, 0.99)** | ref. | 0.95 (0.89, 1.00) | 0.95 (0.90, 1.00) | 0.95 (0.90, 1.01) | 0.97 (0.92, 1.03) |
| Model 5 (+ medical history) | ref. | 0.96 (0.91, 1.02) | 0.98 (0.92, 1.04) | 0.98 (0.92, 1.04) | 0.98 (0.92, 1.04) | ref. | **0.94 (0.89, 0.99)** | 0.96 (0.91, 1.02) | 0.97 (0.92, 1.03) | **0.94 (0.89, 0.99)** | ref. | 0.95 (0.90, 1.00) | 0.95 (0.90, 1.00) | 0.95 (0.90, 1.01) | 0.97 (0.92, 1.03) |
| Sensitivity analysis 1 | ref. | 0.96 (0.91, 1.02) | 0.98 (0.92, 1.04) | 0.98 (0.92, 1.04) | 0.98 (0.92, 1.04) | ref. | **0.94 (0.89, 0.99)** | 0.96 (0.91, 1.02) | 0.97 (0.92, 1.03) | **0.93 (0.88, 0.99)** | ref. | 0.95 (0.90, 1.00) | 0.95 (0.90, 1.00) | 0.95 (0.90, 1.01) | 0.97 (0.92, 1.03) |
| Sensitivity analysis 2 | ref. | 0.95 (0.90, 1.01) | 0.97 (0.91, 1.03) | 0.97 (0.91, 1.03) | 0.97 (0.91, 1.03) | ref. | **0.94 (0.89, 0.99)** | 0.98 (0.92, 1.03) | 0.99 (0.93, 1.04) | 0.94 (0.89, 1.00) | ref. | 0.95 (0.90, 1.00) | 0.94 (0.89, 1.00) | 0.95 (0.90, 1.00) | 0.96 (0.91, 1.01) |
| Sensitivity analysis 3 | ref. | 0.96 (0.91, 1.03) | 0.99 (0.93, 1.06) | 0.98 (0.92, 1.05) | 0.98 (0.92, 1.05) | ref. | 0.94 (0.88, 1.00) | 0.96 (0.91, 1.02) | 0.96 (0.91, 1.02) | 0.94 (0.88, 1.00) | ref. | **0.94 (0.88, 0.99)** | **0.93 (0.88, 0.99)** | 0.95 (0.89, 1.01) | 0.96 (0.91, 1.02) |
| Neurodegenerative disease | | | | | | | | | | | | | | | |
| Population§ | 24902 | 24902 | 24902 | 24902 | 24902 | 24785 | 24785 | 24785 | 24785 | 24785 | 24857 | 24856 | 24856 | 24856 | 24857 |
| No. of events | 397 (1.6%) | 380 (1.5%) | 372 (1.5%) | 356 (1.4%) | 416 (1.7%) | 320 (1.3%) | 394 (1.6%) | 411 (1.7%) | 387 (1.6%) | 399 (1.6%) | 413 (1.7%) | 382 (1.5%) | 373 (1.5%) | 387 (1.6%) | 363 (1.5%) |
| Hill number* | 2.1 (1.0, 2.4) | 2.5 (2.4, 2.5) | 2.6 (2.5, 2.6) | 2.6 (2.6, 2.7) | 2.8 (2.7, 3.6) | 1.6 (1.0, 1.7) | 1.8 (1.7, 1.9) | 1.9 (1.9, 2.0) | 2.0 (2.0, 2.0) | 2.1 (2.0, 3.0) | 1.1 (1.0, 1.2) | 1.4 (1.2, 1.5) | 1.6 (1.5, 1.7) | 1.8 (1.7, 1.9) | 2.0 (1.9, 2.0) |
| HR (95% CI) | | | | | | | | | | | | | | | |
| Model 1 (minimally adjusted) | ref. | 0.91 (0.78, 1.06) | 0.91 (0.78, 1.07) | **0.84 (0.72, 0.99)** | 0.92 (0.79, 1.08) | ref. | 1.09 (0.94, 1.27) | 1.07 (0.92, 1.24) | 1.06 (0.91, 1.23) | 1.06 (0.92, 1.23) | ref. | 0.89 (0.77, 1.02) | **0.84 (0.73, 0.97)** | **0.86 (0.74, 0.99)** | **0.79 (0.69, 0.91)** |
| Model 2 (+ demographics) | ref. | 0.91 (0.79, 1.06) | 0.91 (0.78, 1.07) | **0.85 (0.72, 0.99)** | 0.92 (0.79, 1.08) | ref. | 1.11 (0.95, 1.29) | 1.07 (0.92, 1.24) | 1.07 (0.92, 1.24) | 1.08 (0.93, 1.26) | ref. | 0.90 (0.78, 1.04) | **0.86 (0.74, 0.99)** | 0.87 (0.76, 1.00) | **0.80 (0.69, 0.92)** |
| Model 3 (+ lifestyle) | ref. | 0.91 (0.78, 1.07) | 0.92 (0.79, 1.08) | 0.85 (0.73, 1.01) | 0.92 (0.79, 1.08) | ref. | 1.16 (0.99, 1.35) | 1.11 (0.96, 1.30) | 1.10 (0.94, 1.28) | 1.12 (0.97, 1.31) | ref. | 0.91 (0.79, 1.05) | **0.86 (0.75, 0.99)** | 0.87 (0.76, 1.01) | **0.81 (0.70, 0.94)** |
| Model 4 (+ diet) | ref. | 0.92 (0.79, 1.08) | 0.93 (0.80, 1.10) | 0.86 (0.73, 1.02) | 0.93 (0.79, 1.09) | ref. | 1.17 (1.01, 1.36) | 1.12 (0.97, 1.31) | 1.11 (0.95, 1.29) | 1.14 (0.98, 1.33) | ref. | 0.92 (0.79, 1.05) | 0.87 (0.75, 1.00) | 0.89 (0.77, 1.02) | **0.82 (0.71, 0.96)** |
| Model 5 (+ medical history) | ref. | 0.93 (0.80, 1.08) | 0.94 (0.80, 1.10) | 0.86 (0.73, 1.02) | 0.93 (0.79, 1.09) | ref. | 1.17 (1.00, 1.36) | 1.12 (0.96, 1.30) | 1.10 (0.94, 1.28) | 1.15 (0.99, 1.34) | ref. | 0.91 (0.79, 1.05) | 0.87 (0.75, 1.00) | 0.89 (0.77, 1.02) | **0.82 (0.71, 0.95)** |
| Sensitivity analysis 1 | ref. | 0.93 (0.80, 1.08) | 0.94 (0.80, 1.10) | 0.86 (0.73, 1.02) | 0.93 (0.79, 1.09) | ref. | 1.17 (1.00, 1.36) | 1.12 (0.96, 1.30) | 1.10 (0.94, 1.28) | 1.15 (0.99, 1.34) | ref. | 0.92 (0.79, 1.05) | 0.87 (0.75, 1.00) | 0.89 (0.77, 1.02) | **0.82 (0.71, 0.95)** |
| Sensitivity analysis 2 | ref. | 0.92 (0.79, 1.07) | 0.92 (0.79, 1.08) | 0.85 (0.72, 1.00) | 0.92 (0.78, 1.07) | ref. | 1.16 (1.00, 1.35) | 1.11 (0.96, 1.30) | 1.10 (0.94, 1.28) | 1.15 (0.99, 1.34) | ref. | 0.91 (0.79, 1.05) | 0.86 (0.75, 1.00) | 0.88 (0.76, 1.01) | **0.81 (0.70, 0.94)** |
| Sensitivity analysis 3 | ref. | 0.93 (0.79, 1.08) | 0.94 (0.80, 1.11) | 0.86 (0.73, 1.02) | 0.91 (0.77, 1.07) | ref. | 1.16 (0.99, 1.35) | 1.08 (0.92, 1.26) | 1.09 (0.93, 1.27) | 1.13 (0.96, 1.32) | ref. | 0.93 (0.81, 1.08) | 0.89 (0.77, 1.03) | 0.90 (0.78, 1.05) | **0.84 (0.72, 0.98)** |

[1]Hazard ratios (95% CI) for risk of all-cause mortality and major chronic diseases obtained from Cox proportional hazards models with age as the underlying timescale. models considering flavonoid subclasses were additionally individually adjusted for quantity of intake of the specific flavonoid subclass of interest. Model 1, minimally adjusted for: sex, region of residence and number of dietary recalls. Model 2, multivariable adjusted for model 1 plus demographic factors including: ethnicity, BMI, education, social economic status (Townsend deprivation index). Model 3, multivariable adjusted for model 2 plus lifestyle factors including smoking, physical activity and alcohol intake. Model 4, multivariable adjusted for model 3 plus dietary factors including intakes (g/d) of red and processed meat, refined grains, whole grains, sugary drinks, coffee, saturated fatty acids, sodium and (kcal/d) energy. Model 5, multivariable adjusted for model 4 plus medical history including history of diabetes mellitus, hypertension and hypercholesterolemia and for analysis of all-cause mortality, models were further adjusted for prevalent CVD, cancer, respiratory disease, and neurodegenerative disease at baseline. Sensitivity analysis 1: excludes energy intake from Model 5. Sensitivity analysis 2: adjusts for a healthful plant-based diet index in place of other dietary covariates in Model 5 retaining energy intake. Sensitivity analysis 3: excludes participants who had an event in the first 2 years of follow-up using Model 5; case event numbers listed in the table do not apply to this analysis. §Sample sizes differ between analyses due to differing per analysis exclusions. *Intake shows flavonoid quantity (mg/d) and diversity as Hill's effective numbers. Median; min to max [all such values]. Intake quintiles are mutually exclusive.

**Supplementary Table 6.** Quantity of intake of flavonoid-rich foods, selected flavonoid subclasses and risk of selected outcomes in the UK Biobank.[1]

| | Hazard ratio (95% CI) for quantity of intake (adjusted for diversity) | | | | |
|---|---|---|---|---|---|
| | Q1 | Q2 | Q3 | Q4 | Q5 |
| Flavonoid-rich foods | | | | | |
| All-cause mortality | ref. | 0.94 (0.86, 1.02) | 0.95 (0.87, 1.03) | **0.89 (0.82, 0.97)** | 0.95 (0.87, 1.03) |
| Cardiovascular disease | ref. | 0.98 (0.91, 1.06) | 0.95 (0.88, 1.03) | **0.91 (0.84, 0.99)** | 0.98 (0.91, 1.06) |
| Type 2 diabetes | ref. | **0.87 (0.78, 0.97)** | **0.86 (0.77, 0.95)** | **0.81 (0.73, 0.90)** | **0.82 (0.73, 0.91)** |
| Cancer incidence | ref. | 0.99 (0.92, 1.06) | 0.99 (0.93, 1.06) | 0.98 (0.92, 1.05) | 0.97 (0.91, 1.04) |
| Respiratory disease | ref. | **0.93 (0.88, 0.99)** | **0.93 (0.88, 0.98)** | **0.93 (0.88, 0.98)** | 0.97 (0.92, 1.03) |
| Neurodegenerative disease | ref. | 0.86 (0.74, 1.00) | 0.91 (0.79, 1.06) | 0.91 (0.79, 1.05) | 0.86 (0.74, 1.00) |
| Flavan-3-ols[†] | | | | | |
| All-cause mortality | ref. | **0.85 (0.78, 0.93)** | **0.88 (0.81, 0.96)** | **0.88 (0.81, 0.95)** | **0.86 (0.79, 0.94)** |
| Type 2 diabetes incidence | ref. | 0.92 (0.82, 1.02) | 0.90 (0.81, 1.00) | **0.85 (0.77, 0.95)** | **0.80 (0.72, 0.89)** |
| Cancer incidence | ref. | 1.02 (0.95, 1.09) | 0.99 (0.92, 1.05) | 0.99 (0.93, 1.06) | 0.95 (0.89, 1.02) |
| Flavanones | | | | | |
| All-cause mortality | ref. | 0.98 (0.90, 1.06) | 0.94 (0.86, 1.02) | 0.95 (0.87, 1.03) | 1.00 (0.92, 1.09) |
| Cancer incidence | ref. | 1.00 (0.94, 1.07) | 0.95 (0.89, 1.02) | 0.93 (0.87, 1.00) | 0.97 (0.91, 1.04) |
| Respiratory disease incidence | ref. | 0.98 (0.92, 1.03) | 0.95 (0.90, 1.01) | **0.91 (0.86, 0.97)** | 0.96 (0.90, 1.01) |
| Flavones | | | | | |
| Type 2 diabetes incidence | ref. | 0.99 (0.90, 1.10) | **0.89 (0.80, 0.99)** | **0.84 (0.75, 0.94)** | **0.87 (0.77, 0.97)** |
| Neurodegenerative disease incidence | ref. | 0.97 (0.84, 1.12) | 0.99 (0.86, 1.14) | 0.90 (0.78, 1.05) | 0.85 (0.73, 1.00) |

[1]Hazard ratios (95% CI) for risk of all-cause mortality and major chronic diseases obtained from Cox proportional hazards models with age as the underlying timescale. All models considering quantity of servings of flavonoid-rich food intake were adjusted for diversity of servings of the same flavonoid-rich foods. All models considering quantity of flavonoid subclasses were additionally individually adjusted for diversity of intake of the specific flavonoid subclass of interest. All models were adjusted for sex, region of residence, number of dietary recalls, ethnicity, BMI, education, social economic status (Townsend deprivation index), smoking, physical activity, alcohol intake, and intakes (g/d) of red and processed meat, refined grains, whole grains, sugary drinks, coffee, saturated fatty acids, sodium and (kcal/d) energy and history of diabetes mellitus, hypertension and hypercholesterolemia and for analysis of all-cause mortality, models were further adjusted for prevalent CVD, cancer, respiratory disease, and neurodegenerative disease at baseline. Ref, reference.

[†]Includes monomers, proanthocyanidins and theaflavins/thearubigins.

**Supplementary Table 7.** Sensitivity analysis for quantity and diversity of dietary flavonoid intake and the risk of all-cause mortality and major chronic disease in the UK Biobank.[1]

| | Quantity of total flavonoid intake | | | | | Diversity of total flavonoid intake | | | | |
|---|---|---|---|---|---|---|---|---|---|---|
| | **Q1** | **Q2** | **Q3** | **Q4** | **Q5** | **Q1** | **Q2** | **Q3** | **Q4** | **Q5** |
| All-cause mortality | | | | | | | | | | |
| Sensitivity analysis 1 | ref. | **0.84 (0.78, 0.92)** | **0.85 (0.78, 0.92)** | **0.83 (0.76, 0.90)** | **0.84 (0.77, 0.92)** | ref. | 0.99 (0.91, 1.07) | **0.89 (0.82, 0.97)** | 0.98 (0.90, 1.07) | **0.86 (0.78, 0.94)** |
| Sensitivity analysis 2 | ref. | **0.85 (0.78, 0.92)** | **0.85 (0.78, 0.93)** | **0.84 (0.76, 0.91)** | **0.85 (0.78, 0.94)** | ref. | 1.00 (0.92, 1.08) | **0.91 (0.83, 0.99)** | 0.99 (0.91, 1.09) | **0.87 (0.79, 0.96)** |
| Sensitivity analysis 3 | ref. | **0.85 (0.78, 0.93)** | **0.84 (0.77, 0.92)** | **0.81 (0.74, 0.90)** | **0.83 (0.75, 0.92)** | ref. | 0.98 (0.90, 1.07) | **0.89 (0.81, 0.98)** | 0.95 (0.86, 1.05) | **0.84 (0.76, 0.94)** |
| Cardiovascular disease incidence | | | | | | | | | | |
| Sensitivity analysis 1 | ref. | **0.91 (0.84, 0.98)** | 0.92 (0.85, 1.00) | **0.89 (0.82, 0.97)** | **0.88 (0.81, 0.96)** | ref. | 0.99 (0.92, 1.07) | 0.94 (0.87, 1.01) | 0.94 (0.87, 1.02) | **0.90 (0.82, 0.98)** |
| Sensitivity analysis 2 | ref. | **0.90 (0.83, 0.97)** | **0.91 (0.84, 0.98)** | **0.89 (0.82, 0.96)** | **0.88 (0.81, 0.96)** | ref. | 1.00 (0.93, 1.08) | 0.94 (0.87, 1.02) | 0.95 (0.87, 1.03) | **0.90 (0.83, 0.99)** |
| Sensitivity analysis 3 | ref. | **0.89 (0.82, 0.97)** | **0.89 (0.82, 0.97)** | **0.85 (0.78, 0.92)** | **0.85 (0.77, 0.93)** | ref. | 0.99 (0.91, 1.07) | 0.94 (0.86, 1.02) | 0.92 (0.84, 1.00) | **0.89 (0.81, 0.98)** |
| Type 2 diabetes incidence | | | | | | | | | | |
| Sensitivity analysis 1 | ref. | **0.88 (0.79, 0.98)** | **0.83 (0.75, 0.93)** | **0.79 (0.71, 0.88)** | **0.75 (0.66, 0.84)** | ref. | 0.93 (0.84, 1.03) | 0.93 (0.84, 1.04) | **0.88 (0.79, 0.99)** | **0.80 (0.71, 0.91)** |
| Sensitivity analysis 2 | ref. | **0.88 (0.79, 0.98)** | **0.83 (0.75, 0.93)** | **0.79 (0.71, 0.89)** | **0.75 (0.66, 0.85)** | ref. | 0.93 (0.84, 1.03) | 0.92 (0.83, 1.03) | **0.88 (0.78, 0.99)** | **0.80 (0.70, 0.90)** |
| Sensitivity analysis 3 | ref. | **0.88 (0.79, 0.99)** | **0.83 (0.74, 0.93)** | **0.77 (0.68, 0.87)** | **0.75 (0.66, 0.85)** | ref. | 0.92 (0.82, 1.03) | 0.92 (0.81, 1.03) | 0.89 (0.78, 1.00) | **0.77 (0.67, 0.89)** |
| Cancer incidence | | | | | | | | | | |
| Sensitivity analysis 1 | ref. | 0.96 (0.90, 1.03) | 0.95 (0.89, 1.01) | 0.98 (0.92, 1.05) | **0.92 (0.86, 0.99)** | ref. | 0.95 (0.89, 1.01) | **0.92 (0.86, 0.98)** | 0.97 (0.91, 1.04) | 0.93 (0.86, 1.00) |
| Sensitivity analysis 2 | | 0.96 (0.90, 1.03) | 0.95 (0.88, 1.01) | 0.98 (0.91, 1.05) | 0.92 (0.86, 1.00) | | 0.95 (0.89, 1.01) | **0.91 (0.85, 0.97)** | 0.96 (0.90, 1.03) | **0.92 (0.85, 0.99)** |
| Sensitivity analysis 3 | | 0.93 (0.86, 1.00) | **0.91 (0.84, 0.98)** | 0.94 (0.87, 1.02) | **0.88 (0.81, 0.95)** | ref. | 0.93 (0.86, 1.00) | **0.89 (0.82, 0.96)** | 0.93 (0.86, 1.01) | **0.87 (0.79, 0.94)** |
| Respiratory disease incidence | | | | | | | | | | |
| Sensitivity analysis 1 | ref. | **0.87 (0.83, 0.92)** | **0.88 (0.84, 0.94)** | **0.87 (0.82, 0.92)** | **0.91 (0.86, 0.97)** | ref. | **0.93 (0.88, 0.99)** | **0.91 (0.86, 0.96)** | **0.93 (0.88, 0.99)** | **0.92 (0.86, 0.98)** |
| Sensitivity analysis 2 | ref. | **0.87 (0.82, 0.92)** | **0.88 (0.83, 0.93)** | **0.87 (0.82, 0.92)** | **0.92 (0.86, 0.98)** | ref. | **0.93 (0.88, 0.99)** | **0.91 (0.86, 0.96)** | **0.94 (0.88, 0.99)** | **0.92 (0.86, 0.98)** |
| Sensitivity analysis 3 | ref. | **0.85 (0.80, 0.90)** | **0.85 (0.80, 0.90)** | **0.83 (0.78, 0.89)** | **0.88 (0.82, 0.94)** | ref. | **0.92 (0.87, 0.98)** | **0.88 (0.83, 0.94)** | **0.91 (0.85, 0.97)** | **0.89 (0.83, 0.95)** |
| Neurodegenerative disease incidence | | | | | | | | | | |
| Sensitivity analysis 1 | ref. | **0.82 (0.71, 0.95)** | 0.90 (0.78, 1.04) | 0.87 (0.75, 1.01) | **0.80 (0.68, 0.94)** | ref. | 1.12 (0.96, 1.30) | 1.17 (1.00, 1.36) | 1.12 (0.95, 1.31) | 1.07 (0.90, 1.27) |
| Sensitivity analysis 2 | ref. | **0.81 (0.70, 0.93)** | 0.88 (0.76, 1.02) | **0.85 (0.73, 0.99)** | **0.78 (0.66, 0.93)** | ref. | 1.13 (0.97, 1.31) | 1.19 (1.02, 1.39) | 1.14 (0.96, 1.34) | 1.09 (0.91, 1.30) |
| Sensitivity analysis 3 | ref. | **0.81 (0.70, 0.94)** | 0.88 (0.76, 1.03) | 0.87 (0.75, 1.02) | **0.80 (0.67, 0.94)** | ref. | 1.12 (0.96, 1.31) | 1.20 (1.02, 1.41) | 1.15 (0.97, 1.36) | 1.09 (0.91, 1.30) |

[1]Hazard ratios (95% CI) for risk of all-cause mortality and major chronic diseases obtained from Cox proportional hazards models with age as the underlying timescale. All models considering diversity of flavonoid intake were adjusted for quantity of flavonoid intake and vice versa. Sensitivity analysis 1: excludes energy intake from Model 5. Sensitivity analysis 2: adjusts for a healthful plant-based diet index in place of other dietary covariates in Model 5 retaining energy intake. Sensitivity analysis 3: excludes participants who had an event in the first 2 years of follow-up using Model 5.

**Supplementary Table 8**. Definitions of disease history used in the current study.

| | **ICD-9** | **ICD-10** | **Self-reported fields** |
|---|---|---|---|
| Cardiovascular disease | 410-412, 414, 433, 434, 443.9 | I20-I25, I63 and I70-I74 | 20002(1075, 1074, 1583, 1067, 1087, 1088) |
| Diabetes | 250 | E10-E14 | 20002(1220, 1222, 1223), 100045(6177[3], 6153[3])) |
| Hypertension | 401.9 | I10 | 20002(1072, 1065), 100045(6177[2], 6153[2]) |
| Cancer | 140-208, excluding non-melanoma skin cancer: 173 | C00-C97, excluding non-melanoma skin cancer: C44 | 20001(All cancer codes excluding: 1060) |
| Respiratory disease | 460-519 | J09-J98, I26 and I27 | 20002(1111, 1112, 1113, 1114, 1115, 1117, 1120, 1121, 1122, 1126) |
| Neurodegenerative disease | 331 | F00-03, G12.2, G20, G21, G23.1-23.3, G23.8, G23.9, G30 and G31 | 20002(1263, 1258, 1259, 1262) |
| High cholesterol | Nil | Nil | 20002(1473), 100045(6177[1], 6153[1])) |

Abbreviations: ICD, International Classification of Diseases.

## Supplementary Methods

### Explanation and elaboration: Use of effective numbers to describe flavonoid diversity

The purpose of using Hill's effective numbers is to convert Shannon's nonlinear score into an interpretable metric which describes diversity[1]. In the context of flavonoids, by using this method, we account for 1) the number (i.e., variation) of consumed flavonoids (wherein consumption of more different types of flavonoids contributes to greater diversity) and 2) the relative abundance (i.e., distribution) of the consumed flavonoids (wherein those compounds contributing less or minor intakes relative to other flavonoid types are weighted less). By using Shannon's equation with Hill numbers, we create a new variable which combines the two traits of variation and distribution. This variable is a hypothetical construct, showing the number of different types of flavonoids which would need to be consumed in a specific proportional makeup, to meet the same relative diversity as the diet under study, which we term the "effective number of flavonoids" and wherein a higher value indicates a wider diversity. Effective numbers are often explained in the context of holding one trait constant (usually distribution) while changing the other (usually variation). For example, starting at the effective number of one (or no diversity), when only one type of flavonoid is consumed, a diet with an effective number of exactly 10 (representing a diet as diverse as 10-equally consumed flavonoids) is twice as diverse as a diet with an effective number of exactly 5 (or a diet as diverse as 5-equally consumed flavonoids). On the other hand, a diet with an effective number of 3.8, is still more diverse than a diet with an effective number of 3.2, but these two diets now represent a diet which is as diverse as 3 compounds consumed in equal proportions, plus the fraction of variation and distribution which is contributed by another compound consumed in an unequal amount. Because effective numbers are hypothetical constructs, it is possible that a diet low in variation, but good proportional makeup can have an effective number which is the same as a diet high in variation but poor in proportional makeup. Hill numbers do not show us the actual underlying dietary intakes (or at least, probably not in most cases), rather they simply provide us with an intuitively understandable scale, deriving a hypothetical number of flavonoid types which would need to be consumed in a specific proportional way, in order to describe the relative underlying diversity of the diet under study. The Shannon equation and Hill numbers produce a measure of diversity that is relative and independent from quantity of flavonoid intake, such that it is possible that two individuals can have exactly the same diversity score, yet one of them may consume, for example, a three-fold higher quantity of flavonoids. Therefore, following statistical adjustment for quantity of flavonoid consumption, it is possible to study the independent benefit of diversity of flavonoid intake.

## Supplementary References

1. Jost, L. Entropy and diversity. *Oikos* 113, 363–375 (2006).
