# Supplementary material for: High diversity of dietary flavonoid intake is associated with a lower risk of all-cause mortality and major chronic diseases
Source: Nat Food. 2025 Jun 2;6(7):668–80. doi: 10.1038/s43016-025-01176-1 (PMC12283405; doi:10.1038/s43016-025-01176-1)
Supplement: Supplementary file 2 — Reporting Summary [file 43016_2025_1176_MOESM2_ESM.pdf]

nature portfolio

Corresponding author(s): Aedin Cassidy; Tilman Kuhn

Last updated by author(s): April 10 2025

Reporting Summary

Nature Portfolio wishes to improve the reproducibility of the work that we publish. This form provides structure for consistency and transparency in reporting. For further information on Nature Portfolio policies, see our Editorial Policies and the Editorial Policy Checklist.

Statistics

For all statistical analyses, confirm that the following items are present in the figure legend, table legend, main text, or Methods section.

n/a	Confirmed	
☐	☒	The exact sample size (*n*) for each experimental group/condition, given as a discrete number and unit of measurement
☐	☒	A statement on whether measurements were taken from distinct samples or whether the same sample was measured repeatedly
☐	☒	The statistical test(s) used AND whether they are one- or two-sided<br>*Only common tests should be described solely by name; describe more complex techniques in the Methods section.*
☐	☒	A description of all covariates tested
☐	☒	A description of any assumptions or corrections, such as tests of normality and adjustment for multiple comparisons
☐	☒	A full description of the statistical parameters including central tendency (e.g. means) or other basic estimates (e.g. regression coefficient) AND variation (e.g. standard deviation) or associated estimates of uncertainty (e.g. confidence intervals)
☐	☒	For null hypothesis testing, the test statistic (e.g. *F*, *t*, *r*) with confidence intervals, effect sizes, degrees of freedom and *P* value noted<br>*Give P values as exact values whenever suitable.*
☒	☐	For Bayesian analysis, information on the choice of priors and Markov chain Monte Carlo settings
☒	☐	For hierarchical and complex designs, identification of the appropriate level for tests and full reporting of outcomes
☐	☒	Estimates of effect sizes (e.g. Cohen's *d*, Pearson's *r*), indicating how they were calculated

Our web collection on statistics for biologists contains articles on many of the points above.

Software and code

Policy information about availability of computer code

Data collection	Touch-screen and Online questionnaires, physical measures and clinical laboratory tests were used to collect data.
Data analysis	This study did not generate new or customized code/algorithm. Analyses were undertaken using Stata/IC 14.2 (StataCorp LLC) and R statistics (version 4.2.1) using published libraries and functions. The calculation of diversity was made using the R package 'vegan' version 2.6.2 (https://cran.r-project.org/web/packages/vegan/vegan.pdf).

For manuscripts utilizing custom algorithms or software that are central to the research but not yet described in published literature, software must be made available to editors and reviewers. We strongly encourage code deposition in a community repository (e.g. GitHub). See the Nature Portfolio guidelines for submitting code & software for further information.

Data

Policy information about availability of data

All manuscripts must include a data availability statement. This statement should provide the following information, where applicable:

- Accession codes, unique identifiers, or web links for publicly available datasets
- A description of any restrictions on data availability
- For clinical datasets or third party data, please ensure that the statement adheres to our policy

The UK Biobank dataset used in this study is not publicly available but may be available upon application by bona fide researchers (https://www.ukbiobank.ac.uk/). The UK Nutrient Databank food composition tables are openly accessible (https://www.gov.uk/government/publications/composition-of-foods-integrated-dataset-cofid). The US Department of Agriculture Databases for the Flavonoid (https://agdatacommons.nal.usda.gov/articles/dataset/USDA_Database_for_the_Flavonoid_Content_of_Selected_Foods_Release_3_1_May_2014_/24659802) and proanthocyanin (https://agdatacommons.nal.usda.gov/articles/dataset/USDA_Database_for_the_Proanthocyanidin_Content_of_Selected_Foods_-_2004/25060832) contents in foods are openly accessible.

Human research participants

Policy information about studies involving human research participants and Sex and Gender in Research.

Reporting on sex and gender	All analyses are adjusted for self-reported sex.
Population characteristics	The characteristics of the study participants are described in Table 1.
Recruitment	UK Biobank is a prospective cohort study. Between 2006 and 2010, >500,000 male and female adults, aged 40–69 years old were enrolled. Participants attended one of 22 assessment centers located across England, Scotland, and Wales, where they undertook a comprehensive baseline assessment, completing questionnaires and physical measures, and provided biological samples.
Ethics oversight	The UK Biobank study received ethical approval by the NHS North West Multi-Centre Research Ethics Committee (Ref. 11/NW/0382). All participants provided informed consent.

Note that full information on the approval of the study protocol must also be provided in the manuscript.

Field-specific reporting

Please select the one below that is the best fit for your research. If you are not sure, read the appropriate sections before making your selection.

☒ Life sciences ☐ Behavioural & social sciences ☐ Ecological, evolutionary & environmental sciences

For a reference copy of the document with all sections, see nature.com/documents/nr-reporting-summary-flat.pdf

Life sciences study design

All studies must disclose on these points even when the disclosure is negative.

Sample size	We retrieved all available data from the UK Biobank of 502,236 participants, before applying the exclusion criteria below.
Data exclusions	For the current analysis, we excluded participants who withdrew their consent during follow-up or who completed less than two 24-hour dietary assessments without plausible energy intakes (<800 or >4200 kilocalories/day for men and <500 or >3500 kilocalories/day for women) (Supplementary Figure 1). Additionally, for the respective outcomes of interest, we excluded participants with prevalent CVD, diabetes, hypertension, cancer, respiratory disease, or neurodegenerative disease, prior to the last date of dietary assessment (Supplementary Table 1). Lastly, because Shannon's equation requires intake of at least one kind of flavonoid compound, those with zero total flavonoid intake were excluded, and then, depending on the exposure of interest (e.g., flavonoid-rich foods or intra-subclass diversity etc.,), participants with zero intake of flavonoid-rich foods or specific subclasses were excluded on a per analysis basis, because the collective exclusion at the flavonoid-rich food or intra-subclass level would bias diversity of other levels (e.g., compounds [Supplementary Figure 1]).
Replication	Among ~120,000 UK Biobank Participants, we repeated analysis, using 5 models of adjustment in addition to 3 sensitivity analyses.
Randomization	Not relevant because this is an observational cohort study
Blinding	Not relevant because this is an observational cohort study.

Reporting for specific materials, systems and methods

We require information from authors about some types of materials, experimental systems and methods used in many studies. Here, indicate whether each material, system or method listed is relevant to your study. If you are not sure if a list item applies to your research, read the appropriate section before selecting a response.

Materials & experimental systems

n/a	Involved in the study	
☒	☐	Antibodies
☒	☐	Eukaryotic cell lines
☒	☐	Palaeontology and archaeology
☒	☐	Animals and other organisms
☐	☒	Clinical data
☒	☐	Dual use research of concern

Methods

n/a	Involved in the study	
☒	☐	ChIP-seq
☒	☐	Flow cytometry
☒	☐	MRI-based neuroimaging

Clinical data

Policy information about clinical studies

All manuscripts should comply with the ICMJE guidelines for publication of clinical research and a completed CONSORT checklist must be included with all submissions.

Clinical trial registration	Not relevant because this is an observational cohort study.
Study protocol	Sudlow C, Gallacher J, Allen N, et al. UK biobank: an open access resource for identifying the causes of a wide range of complex diseases of middle and old age. PLoS medicine 2015: 12(3): e1001779.
Data collection	Participants attended one of 22 assessment centers where they undertook a comprehensive baseline assessment, completing questionnaires and physical measures, and provided biological samples. Following this, participants completed web-based 24-h dietary assessments (the Oxford WebQ) issued on five separate occasions between 2009 and 2012.
Outcomes	The outcomes in the current study were all-cause mortality and incidence (first-time fatal or non-fatal events) of CVD, T2DM, total cancer, respiratory disease, and neurodegenerative disease. Date of death was obtained from death certificates held by the National Health Service Information Centre (England and Wales) and the National Health Service Central Register Scotland (Scotland). Dates and causes of hospital admissions were identified through record linkage to Health Episode Statistics (England), the Patient Episode Database (Wales) and the Scottish Morbidity Records (Scotland) as well as the National Cancer Registries (England, Scotland, and Wales). Incident outcomes were defined as a hospital admission or death identified through primary or secondary diagnosis codes using International Classification of Diseases, Tenth Revision (ICD-10) as follows: CVD (I20-I25, I63 and I70-I74), T2DM (E11), cancer (C00-C97, excluding non-melanoma skin cancer: C44), respiratory disease (J09-J98, I26 and I27) and neurodegenerative disease (F00-03, G12.2, G20, G21, G23.1-23.3, G23.8, G23.9, G30 and G31). Hospital admissions follow-up data for CVD, T2DM, respiratory disease and neurodegenerative disease were available until 31st October 2022 for England, 31st August 2022 for Scotland, and 31st May 2022 for Wales. Hospital admissions follow-up data for cancer were available until 31st December 2016 for Wales, 31st December 2020 for England, and 30th November 2021 for Scotland.
